# Leptomeningeal Metastases in High-Grade Adult Glioma: Development, Diagnosis, Management, and Outcomes in a Series of 34 Patients

**DOI:** 10.3389/fneur.2014.00220

**Published:** 2014-11-03

**Authors:** Christopher Dardis, Kelly Milton, Lynn Ashby, William Shapiro

**Affiliations:** ^1^Department of Neurology, Barrow Neurological Institute, Phoenix, AZ, USA

**Keywords:** glioma, glioblastoma multiforme, leptomeningeal, metastases, Ommaya, intrathecal

## Abstract

**Methods:** Leptomeningeal metastases (LM) in the setting of glioma have often been thought to carry a particularly poor prognosis. We sought to better characterize this phenomenon through a review of patients with glioma seen in our institution over the preceding 10 years. We focus here on 34 cases with LM due to grade III or IV glioma. Over the period in question, we estimate a prevalence of almost 4% in those affected by grade IV tumors.

**Results:** Leptomeningeal spread was present at the time of initial diagnosis in 4 patients. Among the others, LM occurred at the time of first progression of disease in 17. The median time to development of LM (excluding those where it was present at initial diagnosis) was 16.4 months [95% confidence interval (CI) 8.2–43.9]. The median time to further progression of disease following LM was 4.9 months (95% CI 3.1–6.9). Twenty-five patients were known to have died at the time of writing. Thus, median overall survival (OS) was 10.2 months (95% CI 8.8–14.7) following LM. At the time of diagnosis of LM, some form of treatment (chemotherapy and/or radiation vs. no treatment) increased OS (median 11.7 vs. 3.3 months, *p* < 0.001 by log-rank test). Use of radiation therapy (vs. no radiation) also increased OS, although the effect was more modest (7.8 vs. 16.8 months, *p* = 0.07). Higher Karnofsky Performance Status (KPS) at the time of diagnosis of LM was associated with OS (*p* = 0.007, median OS for KPS ≥90 19 months vs. 7.8 for KPS <90). In a two-variable model incorporating the use any treatment (vs. none) and KPS, the latter tended to be a more significant predictor of survival (*p* = 0.22 vs. *p* = 0.06 by likelihood-ratio test). This was also true for radiation (vs. none) and KPS (*p* = 0.27 vs. *p* = 0.02). No significant benefit could be demonstrated for the use of chemotherapy considered alone, either systemic or intrathecal. It should be noted that 4 of 9 patients receiving intrathecal chemotherapy had a ventriculo-peritoneal shunt in place during these injections, which may have reduced its effectiveness.

**Conclusion:** Overall, treatment appears to improve outcomes. We favor maximal treatment, as tolerated, particularly with a KPS of ≥70. Such treatment would typically include radiation to the maximum tolerated dose, concurrent, and adjuvant chemotherapy (preferably with an alkyating agent), in addition to intrathecal treatment.

## 1 Introduction

Malignant glioma (in this series grade ≥grade III) with leptomeningeal metastases (LM, also known as leptomeningeal disease or meningeal gliomatosis) has generally been considered a rare and serious condition. No consensus on treatment currently exists. As far as we are aware, this is the largest case series to date addressing this topic.

There are already a number of case reports and small case series describing various approaches to treatment. One series noted promising results with use of thioTEPA (1), while another suggested that Palliative Care may be preferable to intrathecal chemotherapy (2). One case report suggested that liposomal cytarabine may be of value, with a reported time to progression (TTP) of 6 months (3). In cases, where LM is present at the time of first diagnosis, the use of radiation and temozolomide has been advocated (4).

In the present series, we began by identifying all cases of LM from glial tumors in our institution. By this process, we estimated the prevalence of the condition. We sought to characterize the development of LM and to determine which factors are important in its genesis. We aimed to describe the characteristic findings at the time of diagnosis – clinical, radiographic, and of cerebrospinal fluid (CSF).

Our principal question was whether any of the standard modalities (chemotherapy, including intrathecal, and radiotherapy) proposed for treatment of this condition could be shown to influence TTP or overall survival (OS) following the diagnosis of LM. We also addressed whether the patient’s performance status might be more important than any of the above treatments.

Based on our work, we offer suggestions for diagnosis and treatment of LM. We also place the current work in the context of previous similar studies.

## 2 Materials and Methods

We reviewed the records of our weekly Multidisciplinary Central Nervous System Tumor Conference from 2003 to 2013 (*n* = 12,477) for patients with glioma who had LM. We initially included pediatric cases (i.e., age <18) and grade II tumors (including two cases of ependymoma, one myxopapillary). It has been proposed that ependymoma shares a common precursor with glial tumors (5). On reflection, we decided to focus the review on adult patients with grade III and IV tumors in order to minimize heterogeneity.

This work was approved by the Institutional Review Board at St. Joseph’s Hospital, to which the Barrow Neurological Institute is affiliated.

Clinical information was obtained through a review of the available records at St. Joseph’s Hospital. These included visit notes in addition to radiological and pathological data. Relevant laboratory results were reviewed as well. Imaging characteristics were established through a consensus of two reviewers. In some cases, complete information was unavailable as some patients followed up at outside institutions, either prior to or after their diagnosis. Throughout the chart review, variables considered to have possible value were identified. The complete dataset is available as supplementary material. This shows all of the variables considered herein.

Analysis was performed using R version 3.0.1 (2013-05-16) (6–10). This is open-source software for statistical analysis which has been validated for use in clinical practice (11).

For measures of a proportion (e.g., 5/10), a 95% a confidence interval (CI) is generally given when the measure first appears. Although perhaps unnecessary, we include these confidence intervals to illustrate the uncertainty that arises when dealing with small numbers of patients. These confidence intervals are generally wide given the modest number of observations involved. They are derived using a Jeffreys prior. This is a Bayesian method using a minimally informative prior beta distribution and is considered to have optimal coverage properties for this type of CI (12).

Given the small sample size, a significance or *p*-value of ≤0.1 was considered worth highlighting. As the sample size decreases, *p*-values tend to become larger (unless the null hypothesis is true). Thus, when working with such small numbers of observations, a more generous margin that the typical *p* ≤ 0.05 was felt to be reasonable. This is particularly the case in the context of an exploratory (hypothesis generating) analysis.

The mainstay of survival analysis was the Cox proportional-hazards model. In order to assess the effects of a given predictor, we generally used the log-rank test (LRT). In cases where this was close to the margin of significance, we also looked at the likelihood-ratio (LiRT) and Wald tests. All of these test, the significance of a given predictor and should be concordant.

The magnitude of an effect is given as the hazard ratio (HR). This is the multiplier of the “baseline” risk of progression or death given the effect of a predictor.

The only continuous predictor in the Cox models was age. We considered this to be a linear scale.

In cases where *all* subjects progressed, we also used the Mann–Whitney–Wilcoxon (or Mann–Whitney U) test (two-sided). This is a non-parametric test, which compares groups based on the ordering of a variable (e.g., the time to progression). The results should closely approximate the other tests of significance for a Cox model where no censoring is present.

When comparing the survival of more than two groups (e.g., performance status and treatment considered together), we also used the log-rank test for trend. This is designed to detect *ordered* differences in survival curves; that is, at least one group has a survival advantage compared to the next-worst group (in terms of survival). We also used the standard log-rank test, which estimates whether all curves have equal survival.

With such small numbers, we felt that two variables at most could be included in Cox models. We did not consider interactions.

Our only ordinal variable was the Karnofsky Performance Status (KPS). Here, we used Kendall’s tau (KT) to test for association. This was thought to be fairer than considering KPS as continuous. Clinically, a drop from 80 to 70 cannot be equated with a drop from 70 to 60. Similarly, when using the KPS in Cox models, we preferred to include it as an ordinal scale when possible.

## 3 Results

We initially identified 41 cases; these are shown in Table 1, listed by grade and then by time to death. As above, we excluded those age <18 when LM was diagnosed, those with grade II tumors and those where the grade was uncertain. This left 34 subjects, who are the subject of the remainder of the paper.

**Table 1:** **Summary of patients initially identified (*n* = 41)**.

Age	Gender	Time to LM	Pathology (at time of LM)	Treatment	Time to progression	Time to death	Died?
39	f	24.2	GBM	None	NA	0.1	No
47	m	2.8	GBM	None	NA	0.4	Yes
53	m	5.0	GBM	i.t.+RT	0.8	1.2	NA
58	f	3.9	GBM	None	NA	1.3	Yes
58	m	8.5	GBM	None	NA	1.4	No
15	m	27.1	GBM	None	0.8	2.1	Yes
41	m	14.5	GBM	None	NA	3.3	Yes
20	m	7.9	GBM	i.t. + CT + RT	1.3	4.1	Yes
51	f	16.4	GBM	RT	NA	4.5	NA
42	m	141.3	GBM	CT	5.0	5.9	No
56	m	69.5	GBM	i.t. + CT	NA	6.4	Yes
57	m	5.1	GBM	CT	1.4	7.4	Yes
66	f	5.6	GBM	None	3.9	7.8	Yes
57	f	NA	GBM	CT + RT	2.4	8.8	Yes
59	m	16.3	GBM	sx + i.t.	7.4	9.7	Yes
53	m	7.8	GBM	CT + RT	4.7	9.9	Yes
50	f	8.2	GBM	CT	9.1	10.0	Yes
32	f	5.1	GBM	i.t. + CT	5.3	10.2	Yes
62	m	3.2	GBM	CT	5.5	11.7	Yes
26	m	NA	GBM	NA	NA	13.6	Yes
23	f	45.2	GBM	CT + RT	9.0	14.4	Yes
47	m	0.0	GBM	RT	1.6	14.7	Yes
55	f	NA	GBM	CT + RT	6.3	20.2	Yes
19	f	4.9	GBM	RT	6.9	23.5	Yes
41	m	39.6	GBM	i.t. + RT + CT	4.4	32.3	Yes
31	m	65.4	AOA	None	NA	1.0	No
32	f	91.7	AO	i.t. + RT	1.4	2.2	No
32	f	92.1	AO	i.t.	NA	2.4	No
33	m	18.0	AG	CT	3.0	4.7	Yes
41	m	43.9	AOA	i.t. + CT	NA	6.2	Yes
19	m	10.0	AA	NA	NA	9.1	No
40	m	37.0	AO	CT	6.3	13.5	Yes
58	m	220.8	AO	CT + RT	3.1	18.9	Yes
33	m	48.6	AO	sx + rt + ct	9.4	19.0	Yes
51	f	43.2	AO	CT	22.3	42.0	Yes
41	f	83.4	OA	sx + CT	5.2	5.9	No
51	m	21.6	ME	rt	NA	38.4	No
18	m	NA	EP	CT + RT	62.8	75.0	No
31	f	30.4	NA	NA	NA	1.7	No
35	f	39.7	Astro	CT	16.6	22.5	No
3	m	1.2	Astro	NA	NA	NA	NA

*Sorted by pathologic grade and then by time to death*.

*Times are given in months*.

*Age is at time of first diagnosis*.

*LM, leptomeningeal metastases*.

*Pathology: GBM, glioblastoma multiforme; AOA, anaplastic oligoastrocytoma; AO, anaplastic oligodendroglioma; AG, anaplastic glioma (anaplastic PXA vs. anaplastic ganglioglioma); AA, anaplastic astrocytoma; OA, oligoastrocytoma; ME, myxopapillary ependymoma; EP, ependymoma; astro, astrocytic tumor; grade unknown (grade II, III or IV)*.

*Treatment: i.t., intrathecal (via Ommaya); CT, chemotherapy; sx, surgery; RT, radiotherapy (intensity-modulated radiation therapy and/or stereotactic radiosurgery)*.

*NA, not available*.

These patients ranged in age from 19 to 66 years (median 49) at this time. The primary tumor was glioblastoma multiforme (GBM or grade IV glioma) at this time in 24 of 34 patients (71%, CI 54–84).

### 3.1 From initial diagnosis to development of LM

At the time of initial tumor diagnosis, 4 of 34 (12%, CI 4–26) had LM.

In the others, the TTP from initial diagnosis ranged from 2.8 to 221 months (median 16.4). In this group, LM were present at the time of the first progression of disease in 17 of 30 patients (57%, CI 39–73).

In five cases, the primary disease had progressed from a lower to a higher grade. In four cases, oligodendroglioma (grade II) progressed to the anaplastic form (grade III). Another progressed from oligoastrocytoma (grade II) to GBM (grade IV).

In six cases LM was discovered more than 5 years after the tumor was first diagnosed. These are shown in Table 2.

**Table 2:** **Cases of LM occurring >5 years after initial diagnosis**.

Age	Gender	Location	Pathology (at initial diagnosis)	Pathology (when LM)	Progressions	Time to LM (months)
50	m	t+o	GBM	GBM	NA	69.5
28	m	f	AOA	AOA	3	65.4
40	m	f	AO	AO	2	220.8
25	f	f	O	AO	4	91.7
20	f	f	O	AO	3	92.1
30	m	f	O vs. OA	GBM	4	141.3

*Sorted by pathologic grade and then by time to LM*.

*Age – age at initial diagnosis*.

*Location (of primary tumor): t + o, temporal + occipital; f, frontal; Pathology: GBM, glioblastoma multiforme; AOA, anaplastic oligoastrocytoma; AO, anaplastic oligodendroglioma; O, oligodendroglioma; O vs. OA, oligodendroglioma vs. oligoastrocytoma; Progressions, number of progressions (after intial treatment) before LM diagnosed. These were diagnosed clinically and prompted a change in treatment*.

*NA, not available*.

In one-variable Cox models, advancing age was associated with earlier progression (10-year increase, HR = 1.6, *p* < 0.005).

Cases where the original pathology was GBM were more likely to progress rapidly (HR 5, *p* < 0.0005 by LRT and Wilcoxon test, median 7.9 vs. 48.6 months). These patients were generally older (median 50 vs. 30 years, Wilcoxon *p* = 0.003).

Cases centered in the temporal lobe were also more likely to progress rapidly (HR 3.8, *p* = 0.01 by LRT, *p* = 0.03 by Wilcoxon test, median 6.7 vs. 45.9 months). These were more likely to be GBM (Wilcoxon *p* = 0.03).

The 1p19q co-deletion was tested in 4 patients. It was present in one case of oligodendroglioma. This case took longer to progress than the others (92.1 months vs. median of 23.5 months), although this did not reach significance.

There was only one patient who received *no* chemotherapy or radiation at the time of diagnosis; this person progressed slightly sooner (14.5 months vs. median of 16.3 months).

The following factors did not reach significance based on the LRT: gender, location in basal ganglia vs. lobar, laterality (left vs. right and unilateral vs. bilateral), MIB-1 (including MIB-1 <10 vs. ≥10%), MGMT via immunohistochemistry (including MGMT < 50% vs. ≥ 50%), extent of surgery (including gross vs. subtotal resection), use of standard radiation (60 Gy intensity-modulated vs. none), and chemotherapy (some vs. none and also temozolomide vs. none). There were two patients who received the radiation sensitizer motexafin in addition to standard radiation; this also did not predict time to LM vs. those receiving standard radiation.

We recognize the shortcomings of immunohistochemistry in determining MGMT status. While this is a widely employed and relatively simple test, methylation-specific PCR remains the “gold standard” when available (13).

### 3.2 Symptoms, signs, and results when LM diagnosed

#### 3.2.1 Symptoms

Symptoms at onset of LM documented in more than one patient are shown in Table 3.

**Table 3:** **Common symptoms at time of LM**.

Symptom	Frequency (%)	95% CI
Headache	53	37–69%
Gait disturbance	29	16–46%
Nausea	21	10–36%
Pain	18	8–33%
Confusion/altered mental status	18	8–33%

*Sorted by frequency*.

*CI, confidence interval*.

The patient’s KPS had a tendency to decrease when LM were diagnosed. In 17 cases, where this metric was recorded before (i.e., within 3 months) and after (i.e., within 1 week) diagnosis it fell from 0 to 60 points (median 10). Drops in KPS of ≥10 (or ≥20) were not related to the prior KPS (KT).

#### 3.2.2 Imaging

Magnetic resonance imaging (MRI) when LM were diagnosed revealed that proximity of tumor to CSF is crucial to spread. Proximity to a lateral ventricle, in particular, was present in 16 of 32 (50%, CI 33–67) cases with information available.

Other putative origins (each present in at least two patients, and presented in order of frequency) were adjacent to the following locations: the cerebral cortex, the superior temporal sulcus, the transverse fissure, and the central sulcus. In some cases, tumor was present in more than one of the aforementioned locations.

MRI of the whole spinal cord was performed in 12 of 34 (35%, CI 21–52) patients and another two had at least some imaging of the cord. In those undergoing imaging of the whole cord, 9 of 12 (75%, CI 47–92) had symptoms suggesting myelopathy. In the remaining three cases, imaging appears to have been undertaken as a screening exam.

Spread of LM was seen on MRI in 28 of 33 cases with information available (85%, CI 70–94). Common sites included the cortex, occurring in 16 cases (48%, CI 32–65), spine in 5 (15%, CI 6–30), and cauda equina in 4 (12%, CI 4–26).

**Figure 1:**
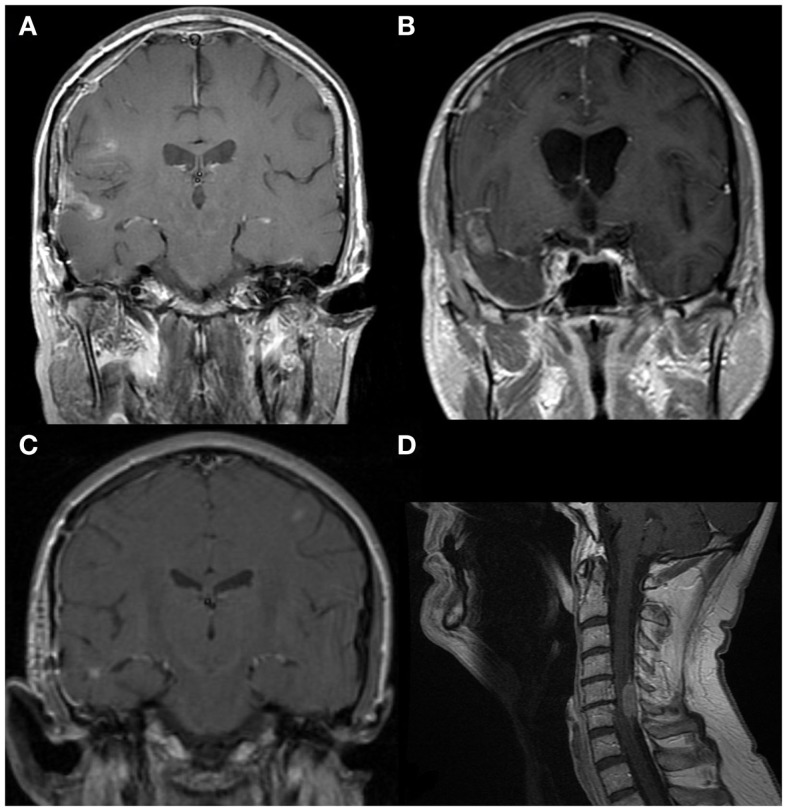
**MRIs (1.5T, T1-weighted, post-contrast)**. **(A)** GBM with local spread to the right superior temporal sulcus. Further spread rostrally along the juxta-cortical leptomeningeal space is present. **(B)** GBM with local spread to the right transverse sulcus. Further spread rostrally along the juxta-cortical leptomeningeal space can also be seen. **(C)** A small focus of GBM in the right temporal lobe with spread to the right middle temporal sulcus, then rostrally along the subarachnoid space on the right. A second focus is seen in the left middle frontal gyrus. This is likely to have spread through the leptomeningeal space. **(D)** GBM with spread to dura at C5–6 and minimal intramedually invasion. Intrathecal chemotherapy is unlikely to penetrate this fully and flow caudally is likely to be impaired.

The spread to spinal cord (and brainstem in some cases) is not surprising given the normal direction of flow of CSF. More striking is that in eleven cases with spread to the cortex, a cortical fissure was the putative origin of cells in seven. The superior temporal sulcus was implicated in five cases and the transverse fissure and superior pre-frontal sulcus in one each. This suggests that flow of CSF from these locations tends to proceed toward the cortex. Examples of this are shown in Figures 1A,B. While intuitive, to our knowledge this phenomenon has not previously been described.

In one unusual case, the tumor appears to have spread from a focus next to the middle temporal sulcus to the contralateral frontal lobe, seen in Figure 1C. Prior to developing this distal spread, the small contralateral focus was the only site of active disease. While intra-parenchymal spread from one location to the other cannot be excluded, spread through the leptomeningeal space appears more plausible.

**Figure 2:**
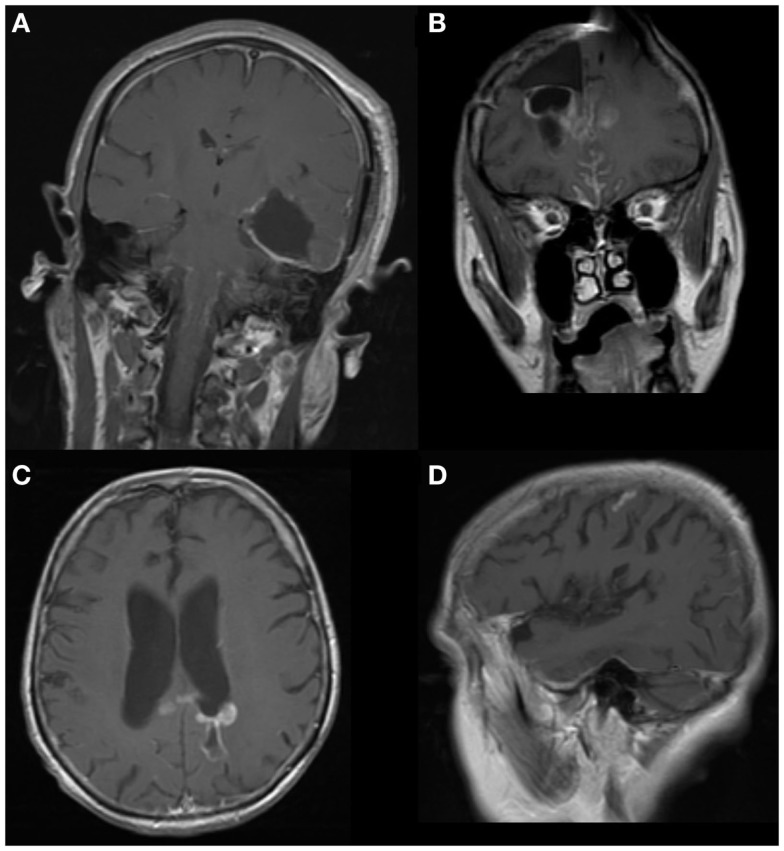
**MRIs (1.5T, T1-weighted, post-contrast)**. **(A)** GBM spread along cerebral cortex and caudally to brainstem. **(B)** GBM with spread to interhemispheric fissures and a new parenchymal focus in the contralateral hemisphere. **(C)** GBM adjacent to left lateral ventricle. **(D)** Leptomeningeal spread from **(C)** to a cortical sulcus on the same side. This was not apparent on axial imaging.

Other representative findings on MRI are shown in Figure 2. In some cases, the findings were subtle as illustrated in Figures 2C,D; these images were taken from the same patient. The presence of tumor next to the lateral ventricle is the probable origin of cells. Spread to a cortical fissure was only apparent on sagittal imaging.

#### 3.2.3 CSF

The result of analysis of CSF at time of diagnosis is shown in Table 4. The initial CSF was taken from an Ommaya reservoir in 5 of 16 cases (31%, CI 13-56). In these cases, the diagnosis had already been made on clinical and radiographic grounds; CSF from a lumbar puncture was not considered essential for confirmation. The CSF from the Ommaya thus served as a “baseline,” which could be used as an adjunct in monitoring response to treatment.

**Table 4:** **CSF results at time of diagnosis**.

	Ommaya	
	No	Yes
WBC	27 ± 13.2	1.0 ± 0.6
Lymph	56.1 ± 13.7	53.3 ± 20.0
MØ	49.2 ± 15.0	NA
Protein	441 ± 181	80.7 ± 21.5
Glucose	65.8 ± 6.4	76.0 ± 7.4
Cytology	0/2	3/9

*Values are given as mean ± standard error*.

*Cytology is no. positive/total no*.

*WBC, white blood cell count (corrected for red cells); Lymph, lymphocytes (% of WBC); MØ, macrophages (%); NA, not available*.

*Protein and glucose measurements are given in mg/dLt*.

The high proportion of macrophages in most samples obtained by lumbar puncture is notable, a finding more classically associated with intracerebral hemorrhage and with fungal infections of the CSF (14).

### 3.3 Initial treatment of LM

Referral for Hospice care took place in 6 of 33 patients (18%, CI 8–34) at the time of diagnosis of LM. These patients tended to have a lower KPS (*p* < 0.01, KT). There was no association with age or pathology (GBM vs. others).

Two patients were lost to follow up following diagnosis. Some form of treatment was attempted in all of the others (*n* = 24), with the exception of one other whose course was complicated by a hip fracture shortly after diagnosis.

**Figure 3:**
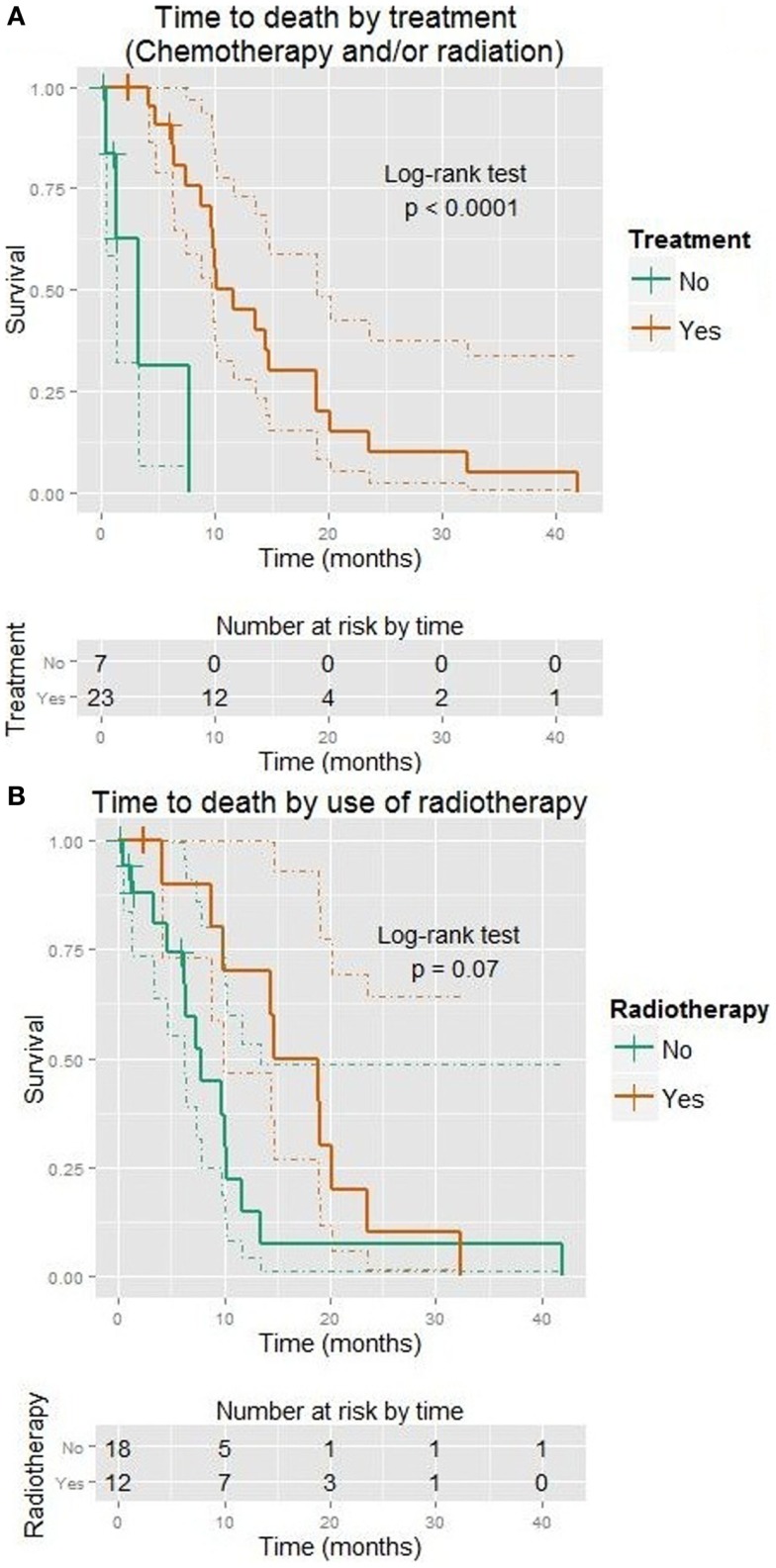
**Kaplan–Meier plots showing time to death (months)**. Dotted lines show 95% confidence intervals for the curves. **(A)** Use of any treatment (vs. none). **(B)** Use of radiation (vs. none).

*Some* form of treatment was clearly beneficial for OS (HR = 0.1, *p* < 0.001, median 11.7 vs. 3.3 months), although no effect could be shown on TTP. This is shown in Figure 3A. Those receiving treatment tended to have a higher KPS (Wilcoxon *p* = 0.02).

#### 3.3.1 Systemic chemotherapy

Systemic chemotherapy was given to 18 of 24 patients (75%, CI 56–89). Of those receiving temozolomide, the standard Stupp protocol was used in 14 (15). Another patient received a “dose dense” regimen of 75 mg/m^2^ daily. In one patient, information on the type of chemotherapy was not available. No effect of systemic chemotherapy could be demonstrated on OS or TTP.

The remaining patients were treated with regimens involving bevacizumab, one in combination with irinotecan, the other with carboplatin. (This latter had already previously been on single-agent bevacizumab).

#### 3.3.2 Intrathecal chemotherapy

Intrathecal chemotherapy via Ommaya was given in 9 of 24 cases (38%, CI 20–57). In five cases Depocyt^®^ (liposomal cytarabine 50 mg q 2 weeks) was administered. Three others received methotrexate (15 mg twice/week) and in one case the type of treatment was not available.

Only three of nine managed at least 6 weeks of treatment before further progression. The longest duration of treatment was a patient on liposomal cytarabine for 14 weeks.

A caveat here is that three of five patients receiving Depocyt and one of three of those on methotrexate had a ventriculo-peritoneal (V-P) shunt in place for at least some of the time that they were on treatment.

Overall, 10 patients had a V-P shunt placed. In six cases, the shunt was placed before LM was diagnosed (median 2.3 months prior to diagnosis with the longest being 44.2 months).

#### 3.3.3 Radiotherapy

Radiotherapy was given to 14 of 24 patients (55%, CI 39–76). In five, focal spinal disease was targeted (one using CyberKnife^®^). In one, radiation of most of the spine was performed. Disease in the brain was targeted with whole-brain radiation in two cases (one of which was stopped early due to rapid deterioration), posterior fossa radiation in one and Gamma Knife^®^ in another.

Radiotherapy improved OS (HR = 0.5, *p* = 0.07, median 16.8 vs. 7.8 months), although no effect could be shown on TTP. This is shown in Figure 3B.

#### 3.3.4 KPS

**Figure 4:**
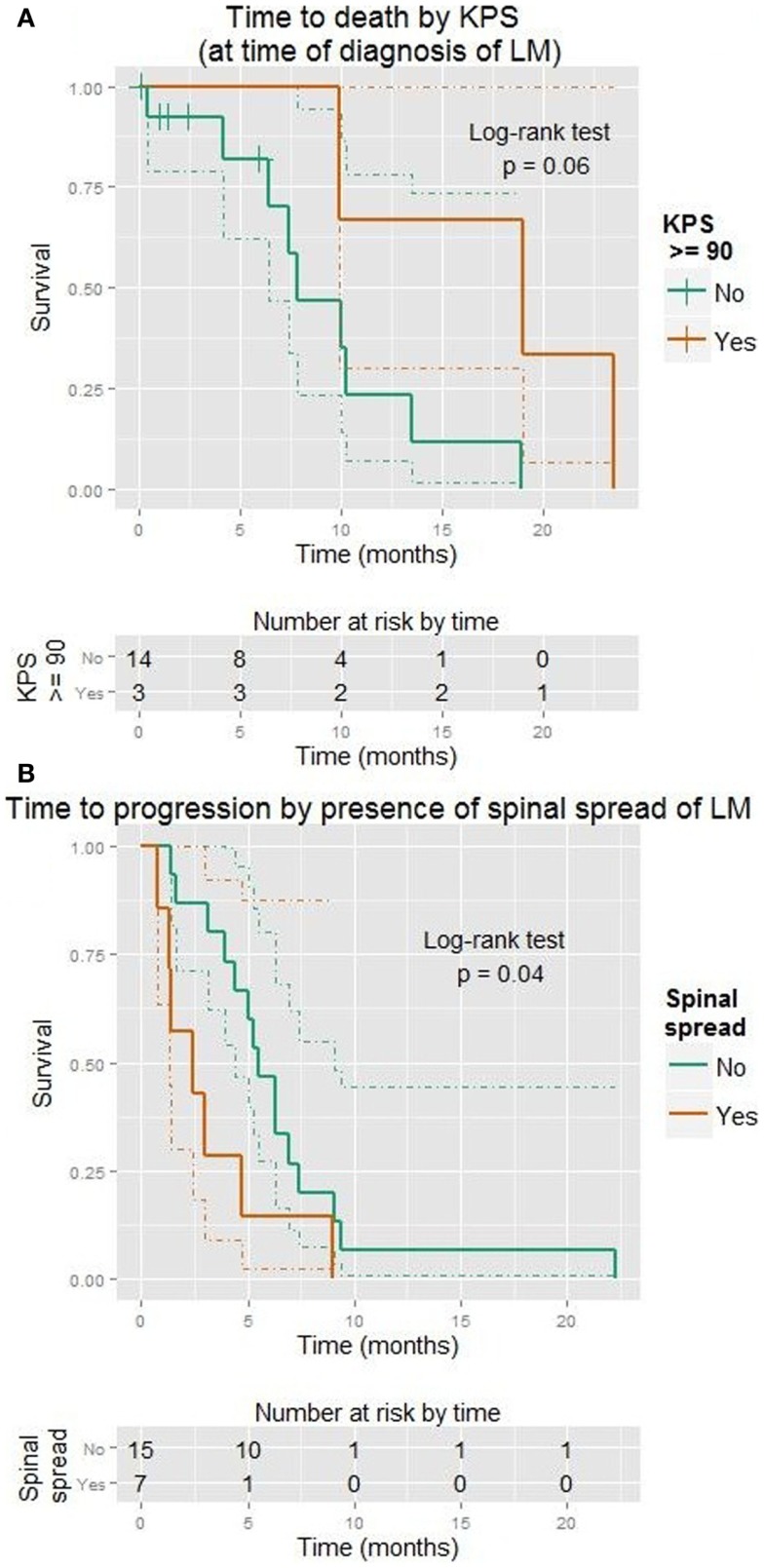
**Kaplan–Meier plots showing time to progression (A) or death (B) in months**. Dotted lines show 95% confidence intervals for the curves. **(A)** KPS ≥90 (vs. <90). **(B)** Spinal spread visible (vs. none).

Karnofsky performance status at time of diagnosis ranged from 60 to 100 (median 70) in those not referred for Hospice care. KPS predicted improved TTP (HR = 0.7 for 5-point increase on linear scale, *p* = 0.04; *p* = 0.09 when considered as ordinal scale). It also predicted OS (HR = 0.7 for 5-point increase on linear scale, *p* = 0.007; *p* = 0.001 as ordinal). A marked difference was established for a KPS of ≥90 (HR = 0.15, *p* = 0.03, median 19 vs. 8 months). This is shown in Figure 4A.

Last KPS measured *prior* to diagnosis of LM also predicted OS (*p* = 0.01 as ordinal).

#### 3.3.5 Spinal metastases

Spread to the spinal cord occurred in 10 cases. There was no association with KPS or presence of GBM. These patients tended to progress more quickly (HR = 2.7, *p* = 0.04, median 2.4 vs. 5.5 months). This is shown in Figure 4B. However, spinal metastases did not affect OS. Most of these patients received radiation (chi-square *p* = 0.004).

#### 3.3.6 Other predictors of progression following initial treatment of LM

Age, gender, and pathology (including GBM vs. others) showed no significant effect on TTP or OS on one-variable Cox models (and Wilcoxon test as appropriate).

#### 3.3.7 Two-variable models

When considering the effects of KPS and radiation on OS, neither the overall model nor either coefficient was significant (*p* = 0.1 by LiRT for model).

**Figure 5:**
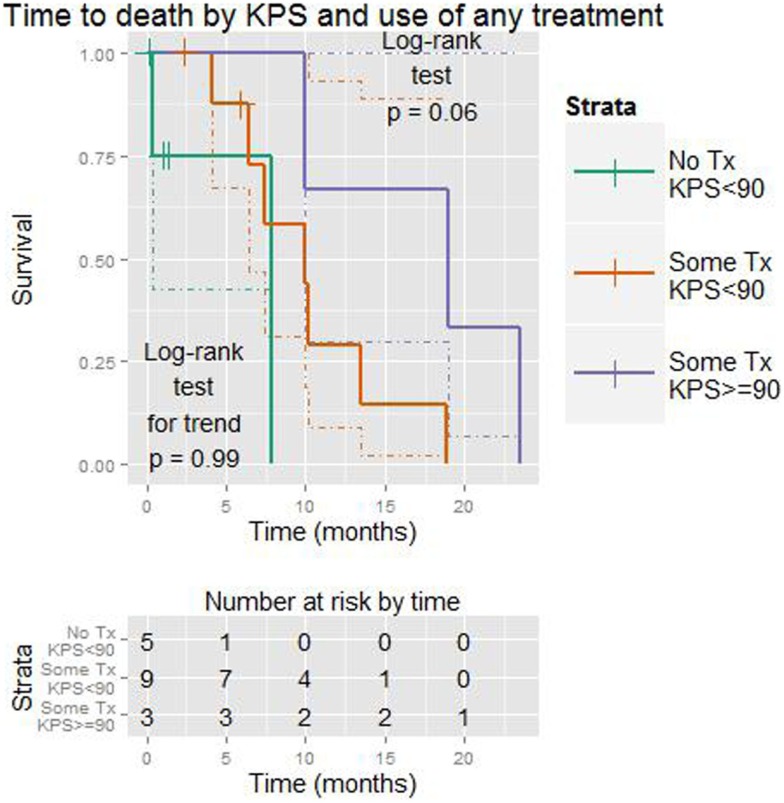
**Kaplan–Meier plot showing time to death (months) with respect to KPS (≥90 vs. <90) and the use of any treatment (vs. none)**. Dotted lines show 95% confidence intervals for the curves. The test for trend is designed to detect *ordered* differences in survival curves.

Karnofsky performance status was a more important factor than the effect of *any* treatment (vs. none) when both were considered together (LiRT *p* = 0.01 vs. *p* = 0.45, Wald *p* = 0.03 vs. *p* = 0.43). A graph of this is shown in Figure 5.

#### 3.3.8 GBM considered alone

A sub-analysis was performed focusing only on those with GBM at the time of diagnosis of LM (*n* = 24). Male patients were more likely to progress rapidly (HR 2.8, LRT = 0.06), although this did not affect OS. Some form of treatment was useful in increasing OS (HR = 0.09, LRT = 0.0003), although treatment was associated with better KPS (Wilcoxon p = 0.03). When considering both together, KPS was a more significant predictor of OS (*p* = 0.01 by LiRT vs. *p* = 0.97).

### 3.4 Treatment following progression of LM

At the time of progression *after* LM, 20 of 29 (69%, CI 51–83) patients with information available received *some* additional treatment. In these 20 patients, the median time to death was 10.2 months (95% CI 9.7–20.2).

Only one additional form of treatment was attempted in 14 of 20 patients (70%, CI 43–86). Nonetheless two cases (one with GBM, the other AO) survived long enough to receive *four* changes in treatment (i.e., they progressed and changed to a new treatment four times).

## 4 Discussion

Our series suggests a benefit to treatment in selected patients with this condition, particularly radiation treatment. The principal weakness of the current study is the heterogeneity of disease severity and of the treatments employed. The wide variation in performance status makes the effects of treatment difficult to discern in such a small sample. This is further complicated by the natural selection bias that occurs when deciding on treatment for such sick patients (Almost 20% were referred for Hospice care at the time of diagnosis.). These same problems make the design of clinical trials for this condition particularly challenging.

### 4.1 Comparisons to other case series

Two similarly sized series have reported results of use of intrathecal chemotherapy for this condition (1, 2). These, along with an earlier series, are summarized in Table 5. It is striking that the median OS time in the earliest series, from 1980, is *better* than our current series, yet only one patient received treatment [with i.t. chemotherapy (16)]. However, in this latter 4 of 12 patients were diagnosed only at autopsy, whereas all of ours had symptoms or signs leading to diagnosis ante mortem.

**Table 5:** **Series reporting survival in patients with LM treated with i.t. chemotherapy**.

Series	*n*	GBM%	CT%	RT%	i.t.%	i.t. type	mPFS	mOS
Dardis (current)	34	71	56	44	28	liposomal cytarabine/MTX	4.9	10.2
Chamberlain (2)	18	44	72	61	100	MTX ⇒ Ara-C ⇒ thioTEPA	3	3.5
Witham et al. (1)	14	64	36	71	100	thioTEPA	NA	10
Yung et al. (16)	12	75	0	0	8	MTX + Ara-C	NA	11.3

*i.t.%, % receiving intrathecal CT; mPFS, median progression-free survival; mOS, median overall survival; MTX, methotrexate; Ara-C, cytarabine (cytosine arabinoside); NA, not available*.

It appears that LM occurring in those with GBM should be regarded as more aggressive than that stemming from lower-grade gliomas. Our patients with GBM had a median OS (from time of diagnosis of LM) of 9.9 vs. 18.9 months for the grade III tumors, although this did not reach significance. This finding is in keeping with the series from Witham et al. (1), which also reported a shorter survival for those with GBM.

Reported cases of spinal metastases from GBM in this setting have already been the subject of a review [*n* = 24 (17)]. In 37 patients with available survival data, the mean survival time was reported to be 3.7 months (range 0.1–12). We had five patients with GBM, LM, and spinal involvement, with a median OS of 9.9 months, which was not significantly different from those without spinal involvement. In the series from Lawton et al. (17), chemotherapy was used in 14% of cases, with radiotherapy in 77%. Intrathecal chemotherapy was not used in any of these cases.

In keeping with earlier series focusing on those with dissemination to the spinal cord, all of our cases had a clear source of communication with CSF (18). Also consistent with data presented in this series, patients with spinal cord involvement experienced a relatively short time to further progression (median 2.4 months in our 7 patients). Another review, (*n* = *19*), focusing on spinal metastases reported a mean OS time of 4.5 months, also shorter than in our series (19).

Radiation with temozolomide has been advocated as the treatment of choice in cases where LM is present at the time of first diagnosis (4). This was based on a review of 15 cases. One patient survived 22 months from diagnosis with this treatment. The 6 patients in our series treated in this way did reasonably well with a median PFS of 4.6 months and median OS of 18.9 months.

### 4.2 Estimates of prevalence

Symptomatic LM has traditionally been considered rare. In the case of GBM, rates of 2–3% have been reported (20, 21). However, symptomatic LM may occur in up to 19% of cases of GBM involving the cerebellum (22). We estimate that our institution sees *c*. 60 cases of newly diagnosed GBM per year. Thus per our calculations, over 10 years *c*. 3.8% of patients experienced this complication, which is broadly in agreement with the above figures.

However, the rate of LM in gliomas may be underestimated as autopsy series report rates in the range of 20–25% (16, 23, 24). It is likely that we are underestimating the true rate, as no autopsies were performed the patients in our series. One series where all patients underwent autopsy reported ante mortem diagnosis in only 8 of 12 patients (16). Generally speaking, the rate of autopsy appears to be falling over time: for example in the USA this decreased from 19 to 9% over the period 1972–2007 (25).

### 4.3 Time to development of LM

The findings that the time to development of LM appears shorter in older patients and in patients with GBM (vs. grade III tumors) are not unexpected. More striking is the association with an initial location in the temporal lobe. We attribute this to its close proximity to the superior temporal sulcus, the transverse fissure, the central sulcus, and the lateral ventricle. These all appear to be common conduits for the dispersion of malignant cells into the CSF.

While time to development of LM was generally short, it is striking that is isolated cases this can occur many years after the initial diagnosis. This phenomenon appears to be associated with evolution of the tumor to a higher pathologic grade.

### 4.4 Suggestions for diagnosis

A summary of recommendations is shown in Box 1.

Box 1 **Diagnosis of LM.**

- Clinical: suspicious symptoms/signs, particularly hydrocephalus
- Radiographic: MRI brain + whole spine with contrast
- CSF: cytology; cell count, protein, glucose

The diagnosis of LM is ultimately clinical. Support is typically sought from imaging and CSF. Occasional cases are diagnosed on the basis of imaging without any new signs or symptoms. Positive CSF cytology, while sufficient, is not essential for diagnosis. In the appropriate context, CSF may increase the “post-test” probability of diagnosis. Establishing “baseline” CSF results at the time of diagnosis is worthwhile as this allows the clinician to monitor the response to treatment. A low threshold for further investigation is warranted once the diagnosis of LM is entertained in the setting of glioma. This is particularly the case where the original tumor location is in close proximity to CSF.

The need for placement of a CSF shunt (particularly where no clear mechanical obstruction is present) should always prompt consideration of LM. In one series of 12 such patients, 10 showed evidence of hydrocephalus (16). Interestingly, in a series of 7 patients with communicating hydrocephalus in the context of GBM, none showed MRI changes typical of LM (26). In this latter series CSF cytology was generally normal, although protein was typically elevated. The authors suggest that initial post-operative radiation may cause fibrosis of the arachnoid granulations leading to blockages by protein released at the time of a second surgery. We saw no evidence of such an association (Fisher’s exact test), suggesting that LM itself is likely to be the more important cause in most patients. Given that *some* form of treatment is valuable for LM, this makes the investigation of hydrocephalus all the more important not to overlook in those with glial tumors.

Intractable nausea/vomiting should lead to a high degree of suspicion of LM (27). We only had three patients with disease affecting the 4th ventricle; two presented with nausea.

Complete spinal imaging (MRI + contrast if possible) should be done when practical. Although the majority of the patients in our series who underwent spinal imaging had symptoms, which suggested spinal involvement, it is likely that some cases of additional asymptomatic disease would thereby be uncovered, which may lead to changes in management. In particular, focal radiation to asymptomatic spinal LM appears a reasonable strategy. According to at least one case report, FDG-PET may also be useful in this setting (28).

### 4.5 Suggestions for treatment

Being a rare complication of an uncommon disease, this problem is unlikely to be the subject of prospective trials without multi-institutional co-operation. For the present, management is individualized and generally guided by retrospective series such as this. A summary of strategies is shown in text Box 2.

Box 2 **Treatment of LM.**

- Radiation
  - Brain: whole-brain 30 Gy; consider focal or additional dosing to posterior fossa
  - Spine (focal): intensity-modulated 40–50 Gy
  - Concurrent daily temozolomide 75 mg/m^2^ as tolerated
- Adjuvant chemotherapy: e.g., alkylator (temozolomide, lomustine) + bevacizumab
- Intrathecal (via Ommaya): typically liposomal cytarabine

Overall, treatment appears to improve outcomes. We favor maximal treatment, as tolerated, particularly when the KPS is ≥70. This would typically involve radiation to the maximum tolerated dose, while avoiding damage to surrounding structures. The dosing is individualized and based primarily on prior radiation exposure. For the spinal cord, focused (e.g., intensity-modulated) approaches are preferred. For first treatment, doses of 40–50 Gy may be employed. As a rule, we prefer to avoid irradiation of the entire spinal cord, given the risk of myelosuppression. For the brain, whole-brain radiation is typically employed, with first doses in *c*. 30 Gy. In individual cases the posterior fossa alone may receive treatment, or be given an additional boost.

The use of radiation does appear to improve OS. However, a low KPS following diagnosis of LM may outweigh *any* intervention, particularly in the case of chemotherapy.

Regarding radiation, focal clumps of cells may impair the flow of CSF, thus limiting the penetration of intrathecal chemotherapy, rendering treatment via Ommaya ineffective. An example of this is offered in Figure 1D. This may be formally assessed using a radioisotope as per (29) although we are not routinely implementing this technique.

Systemic chemotherapy is also individualized and depends primarily on prior treatment. If radiation is to be employed, it appears rational to combine this with an alkylating agent with good CSF penetration, typically temozolomide. Adjuvant systemic treatment will typically involve an escalation or change in approach, e.g., the addition of bevacizumab to temozolomide or for those already on this combination, a change from temozolomide to lomustine.

Toxicities from intrathecal (i.t.) treatment described in the series by Chamberlain (2) were much higher than in our series with 12 of 18 developing aseptic meningitis. The authors’ suggestion of a palliative approach to these patients is understandable in this context. By contrast, Witham et al. (1) suggest their results are promising and that i.t. treatment should be considered for all patients.

Regarding liposomal cytarabine, while no definite benefit can be attributed to its use our series, at least one case report shows some evidence of benefit and it is generally well tolerated (3). The reported TTP of 6 months in the cited case is similar to that of five patients in our series receiving this treatment, who had a median TTP of 4.4 months. This may be started concurrently with radiation.

#### 4.5.1 Certain caveats apply

In our patients receiving liposomal cytarabine with an Ommaya, three of five also had a V-P shunt (located in a lateral ventricle). In such cases, persistence of the instilled agent in CSF is likely to be shorter. Currently, we employ a strategy of turning off the shunt prior to instillation (where possible) and leaving it off for most of 1 day (or longer if possible, typically if the patient is in hospital and can be monitored). Placement of the Ommaya in a basal cistern is another rational approach.

Another consideration should be the inconvenience of travel (for the patient) for an instillation; this can be significant for an agent like methotrexate, which is administered twice/week. For this purpose, we are investigating the possibility of training a caregiver in the use of chemotherapy. Such an approach has already been shown to be practical for intravenous antibiotics (30).

## 5 Conclusion

Leptomeningeal spread of glioma, while often considered calamitous, does not necessarily mean that treatment should be abandoned. The treatments proposed above are relatively benign and in most cases, the benefits are likely to outweigh the side-effects and inconvenience. The patient’s performance status is vital in individualizing decisions.

## Conflict of Interest Statement

The authors declare that the research was conducted in the absence of any commercial or financial relationships that could be construed as a potential conflict of interest.

## Supplementary Material

The Supplementary Material for this article can be found online at http://www.frontiersin.org/Journal/10.3389/fneur.2014.00220/abstract

Data Sheet 1.pdf **Shows the R code used for analysis**.

Data Sheet 2.csv **Shows the dataset from which the results were generated**.

Data Sheet 3.csv **Shows the key to Data Sheet 2 (column names, abbreviations and explanations)**.
