# Supplementary material for: Leptomeningeal Metastases in High-Grade Adult Glioma: Development, Diagnosis, Management, and Outcomes in a Series of 34 Patients
Source: Front Neurol. 2014 Nov 3;5:220. doi: 10.3389/fneur.2014.00220 (PMC4217477; doi:10.3389/fneur.2014.00220)
Supplement: Data Sheet 1.pdf — Shows the R code used for analysis. [file Data_Sheet1.PDF]

```
###-----------------------------------------
###
### setup
###
###-----------------------------------------
###
### binomial priors
library(binom)
### get development version on survMisc
library(devtools)
### temporary install of current version of survMisc 0.4.2
### note this is now available on CRAN
dev_mode(TRUE)
install_github(username="dardisco", repo="survMisc")
### change your lib.loc as applicable
library(survMisc, lib.loc="C:/Users/c/R-dev")
###
### 95% CIs for 1-34/34
###
round(cbind(100*seq(34)/34,
             100*binom.confint(seq(34), 34,
                                conf.level=0.95,
                                methods="bayes")[-1]
            ),
      digits=1)
###
### get data
setwd("c:/Users/c/Documents/a_lepto/a")
d1 <- read.csv("g1.csv", header=TRUE, stringsAsFactors=FALSE)
names(d1)
### remove pediatric cases, ependymoma and low-grade cases
table(d1$pathLmd, useNA="always")
d1 <- subset(d1, subset = pathLmd %in% c("AA", "AG", "AOA", "AOD", "GBM"))
nrow(d1 <- d1[!d1$ageLmd<=18, ])
###
### variables used later
###
### all experienced progression:
### (for model fitting, NA's in predictor
###  variables will exclude cases as required)
d1$e <- rep(1, nrow(d1))
### GBM at initial Dx
d1$GBM <- d1$gradeDx==4
### spinal mets
d1$sm <- grepl("spine", d1$lmdSpread) |
    grepl("cauda", d1$lmdSpread) |
    grepl("L1", d1$lmdSpread) |
    grepl("intradural", d1$lmdSpread)
### any Tx effective
d1$anyTxLmd <- d1$it | d1$ct | d1$rt
### KPS >= 90
d1$kGr90 <- ifelse(d1$kpsLmd >= 90, 1, 0)
###
###-----------------------------------------
### abstract
###-----------------------------------------
###
### time of Dx
###
sum(d1$lmdAtDx)
```

```
sum(d1$lmdFirstProg, na.rm=TRUE)
###
### time to LMD
###
(s1 <- survfit(Surv(ttLmd, e) ~ 1, data=d1[!d1$lmdAtDx, ]))
###
### overall median ttp and ttd
###
(s1 <- survfit(Surv(ttpLmd, e) ~ 1, data=d1))
summary(d1$ttpLmd)
sum(d1$d, na.rm=TRUE)
(s1 <- survfit(Surv(ttlLmd, d) ~ 1, data=d1))
summary(d1$ttlLmd[d1$d==1])
###
### no. died
###
sum(d1$d, na.rm=TRUE)
### time to death
summary(d1$ttlLmd[d1$d==1])
###
### any Tx effective?
###
### ttp - any
(s1 <- survfit(Surv(ttpLmd, e) ~ anyTxLmd, data=d1))
sig(c1 <- coxph(Surv(ttpLmd, e) ~ anyTxLmd, data=d1))
wilcox.test(ttpLmd ~ anyTxLmd, data=d1, exact=FALSE)
### ttd - any
(s1 <- survfit(Surv(ttlLmd, d) ~ anyTxLmd, data=d1))
sig(c1 <- coxph(Surv(ttlLmd, d) ~ anyTxLmd, data=d1))
### ttd - RT
(s1 <- survfit(Surv(ttlLmd, d) ~ rt, data=d1))
sig(c1 <- coxph(Surv(ttlLmd, d) ~ rt, data=d1))
### ttp - KPS
d1$kGr90 <- ifelse(d1$kpsLmd >= 90, 1, 0)
(s1 <- survfit(Surv(ttlLmd, d) ~ kGr90, data=d1))
sig(c1 <- coxph(Surv(ttlLmd, d) ~ kGr90, data=d1))
###
### two-variables
(s1 <- survfit(Surv(ttlLmd, d) ~
               anyTxLmd + kGr90, data=d1))
sig(c1 <- coxph(Surv(ttlLmd, d) ~
             anyTxLmd + kGr90, data=d1))
summary(c1)
###
### on i.t.Tx with shunt
with(d1, table(it, vps))
###
###----------------------------------------
### intro
###----------------------------------------
###
### age
###
summary(d1$ageLmd)
###
### pathology at Dx
###
table(d1$pathDx)
### GBM at Dx
###
s1 <- sum(d1$lmdGbm, na.rm=TRUE)
```

```
s2 <- sum(is.finite(d1$lmdGbm))
c(s1/s2, binom.confint(s1, s2, conf.level = 0.95, methods="bayes")[-1])
###
###-----------------------------------------
### progression to LMD
###-----------------------------------------
###
### Lmd at Dx
s1 <- sum(d1$lmdAtDx, na.rm=TRUE)
s2 <- sum(is.finite(d1$lmdAtDx))
c(s1/s2, binom.confint(s1, s2, conf.level = 0.95, methods="bayes")[-1])
### time to Lmd if not present at Dx
summary(d1$ttLmd[!d1$lmdAtDx==1])
### Lmd not present at time Dx
d2 <- d1[!d1$lmdAtDx, ]
### time to Lmd
summary(d2$ttLmd)
###
### Lmd present at first progression
s1 <- sum(d1$lmdFirstProg, na.rm=TRUE)
### total Lmd not present initially
s2 <- sum(is.finite(d1$lmdFirstProg))
c(s1/s2, binom.confint(s1, s2, conf.level = 0.95, methods="bayes")[-1])
###
### path~ progressed?
s1 <- sum(d1$pathProg, na.rm=TRUE)
s2 <- nrow(d1) - sum(is.na(d1$lmdFirstProg))
c(s1/s2, binom.confint(s1, s2, conf.level = 0.95, methods="bayes")[-1])
xtabs( ~ pathDx + pathLmd, data=d1[d1$pathProg==1, ])
### time to Lmd >5y
addmargins(table(d2$ttLmd[d2$ttLmd>60], d2$pathLmd[d2$ttLmd>60]))
d3 <- d2[d2$ttLmd>60, ]
d4 <- as.matrix(d3[, c("ageDx", "sex", "loc", "pathDx", "pathLmd",
                       "noProg", "ttLmd"
                       )])
rownames(d4) <- rep("", nrow(d4))
colnames(d4) <- c("Age", "Sex", "Location",
                  "Path1", "Path2", "Progressions", "ttLMD")
d4 <- as.data.frame(d4)
d4 <- d4[order(d4$Path1, d4$ttLMD), ]
library(xtable)
x1 <- xtable(d4)
print(x1, include.rownames = FALSE, NA.string = "NA")
###
###-----------------------------------------
### survival analysis - time to LMD
###-----------------------------------------
###
### overall (if not present at time of Dx)
###
(s1 <- survfit(Surv(ttLmd, e) ~ 1, data=d1[!d1$lmdAtDx, ]))
with(d1, summary(ttLmd[!lmdAtDx]))
###
### age
###
(s1 <- survfit(Surv(ttLmd, e) ~ ageDx, data=d1))
(sig(c1 <- coxph(Surv(ttLmd, e) ~ ageDx, data=d1)))
### hazard ratio for 10-year increase
exp(10*coef(c1))
###
### gender
```

```
###
xtabs(ttLmd ~ sex, data=d1)
(s1 <- coxph(Surv(ttLmd, e) ~ sex, data=d1))
sig((c1 <- coxph(Surv(ttLmd, e) ~ sex, data=d1)))
wilcox.test(ttLmd ~ sex, data=d1)
###
### laterality
###
(s1 <- survfit(Surv(ttLmd, e) ~ lat, data=d1))
sig((c1 <- coxph(Surv(ttLmd, e) ~ lat, data=d1)))
### bilateral vs unilateral
d1$bil <- ifelse(d1$lat=="b", 1, 0)
(s1 <- survfit(Surv(ttLmd, e) ~ bil, data=d1))
sig(c1 <- coxph(Surv(ttLmd, e) ~ bil, data=d1))
###
### location
###
(s1 <- survfit(Surv(ttLmd, e) ~ loc, data=d1))
### lobar vs basal ganglia
(s1 <- survfit(Surv(ttLmd, e) ~ sLoc, data=d1))
sig((c1 <- coxph(Surv(ttLmd, e) ~ sLoc, data=d1)))
### frontal vs temporal
d1$ft <- d1$loc
d1$ft[!d1$loc=="f" & !d1$loc=="t"] <- NA
(s1 <- survfit(Surv(ttLmd, e) ~ ft, data=d1))
sig(c1 <- coxph(Surv(ttLmd, e) ~ ft, data=d1))
wilcox.test(ttLmd ~ ft, data=d1)
### check if partially due to path
xtabs( ~ ft + GBM, data=d1)
d1$GBM <- as.numeric(d1$GBM)
d1$ft <- as.numeric(factor(d1$ft))
wilcox.test(GBM ~ ft, data=d1, exact=FALSE)
###
### path
###
(s1 <- survfit(Surv(ttLmd, e) ~ pathDx, data=d1))
sig(c1 <- coxph(Surv(ttLmd, e) ~ pathDx, data=d1))
### cases with >1 observation
d2 <- d1[d1$pathDx=="AOA"|
         d1$pathDx=="GBM"|
         d1$pathDx=="AOD"|d1$pathDx=="OD", ]
d2 <- transform(d2, pathDx = droplevels(as.factor(pathDx)))
(s1 <- survfit(Surv(ttLmd, rep(1, nrow(d2))) ~
               pathDx, data=d2))
sig(x <- coxph(Surv(ttLmd, rep(1, nrow(d2))) ~
             pathDx, data=d2))
### grade
(s1 <- survfit(Surv(ttLmd, e) ~ gradeDx, data=d1))
sig(c1 <- coxph(Surv(ttLmd, e) ~
             as.factor(gradeDx), data=d1))
### GBM vs others
(s1 <- survfit(Surv(ttLmd, e) ~ GBM, data=d1))
sig(c1 <- coxph(Surv(ttLmd, e) ~ GBM, data=d1))
wilcox.test(ttLmd ~ GBM, data=d1)
### check if partially due to age
xtabs( ~ ageDx + GBM, data=d1)
with(d1, c(median(ageDx[GBM==TRUE]), median(ageDx[GBM==FALSE])))
wilcox.test(ageDx ~ GBM, data=d1, exact=FALSE)
###
### histology
###
```

```
names(d1)[11:17]
for (i in 11:17) {
    s1 <- survfit(Surv(ttLmd, e) ~ d1[, i], data=d1)
    c1 <- coxph(Surv(ttLmd, e) ~ d1[, i], data=d1)
    print(names(d1[i]))
    print(s1)
    print(c1)
    cat("\n\n")
}
### 1p19q
xtabs( ~ X1p19q + pathDx, data=d1)
(s1 <- survfit(Surv(ttLmd, e) ~ X1p19q, data=d1))
sig((c1 <- coxph(Surv(ttLmd, e) ~ X1p19q, data=d1)))
wilcox.test(ttLmd ~ X1p19q, data=d1)
###
### surgery
###
(s1 <- survfit(Surv(ttLmd, e) ~ sxDx, data=d1))
sig((c1 <- coxph(Surv(ttLmd, e)~ sxDx, data=d1)))
### GTR vs STR
d1$sxDx[!d1$sxDx=="GTR" & !d1$sxDx=="STR"] <- NA
(s1 <- survfit(Surv(ttLmd, e) ~ sxDx, data=d1))
sig((c1 <- coxph(Surv(ttLmd, e)~ sxDx, data=d1)))
wilcox.test(ttLmd ~ sxDx, data=d1, exact=FALSE)
###
### any Tx at diagnosis
###
with(d1, table(sRtDx))
with(d1, table(ctDx))
d1$anyTxDx <-d1$sRtDx!="none" | d1$ctDx!="none"
(s1 <- survfit(Surv(ttLmd, e) ~ anyTxDx, data=d1))
sig(c1 <- coxph(Surv(ttLmd, e)~ anyTxDx, data=d1))
###
### RT
###
(s1 <- survfit(Surv(ttLmd, e) ~ sRtDx, data=d1))
sig(c1 <- coxph(Surv(ttLmd, e)~ sRtDx, data=d1))
### 60t vs none
d1$n60 <- d1$sRtDx
d1$n60[!d1$sRtDx=="60t" & !d1$sRtDx=="none"] <- NA
(s1 <- survfit(Surv(ttLmd, e) ~ n60, data=d1))
sig(c1 <- coxph(Surv(ttLmd, e)~ n60, data=d1))
### compare RTt and RTtm
d2 <- d1[d1$sRtDx=="60t"|d1$sRtDx=="60tm", ]
d2 <- transform(d2, sRtDx = droplevels(as.factor(sRtDx)))
(s1 <- survfit(Surv(ttLmd, rep(1, nrow(d2))) ~
               sRtDx, data=d2))
sig(c1 <- coxph(Surv(ttLmd, rep(1, nrow(d2))) ~
            sRtDx, data=d2))
wilcox.test(ttLmd ~ sRtDx, data=d2, exact=FALSE)
###
### CT
###
(s1 <- survfit(Surv(ttLmd, e) ~ ctDx, data=d1))
### TMZ vs none
d2 <- d1[d1$ctDx=="TMZ"|d1$ctDx=="none", ]
(s1 <- survfit(Surv(ttLmd, rep(1, nrow(d2))) ~
               ctDx, data=d2))
sig(c1 <- coxph(Surv(ttLmd, rep(1, nrow(d2))) ~
             ctDx, data=d2))
wilcox.test(ttLmd ~ ctDx, data=d2, exact=FALSE)
```

```
###
### duration of CT
###
(s1 <- survfit(Surv(ttLmd, e) ~ mCtDx, data=d1))
sig(c1 <- coxph(Surv(ttLmd, e) ~ mCtDx, data=d1))
###
### no. progressions
###
(s1 <- survfit(Surv(ttLmd, e)~ noProg, data=d1))
sig(c1 <- coxph(Surv(ttLmd, e)~ noProg, data=d1))
###
###----------------------------------------
### descriptives at Dx LMD
###----------------------------------------
###
### symptoms
names(d1)[35:39]
for (i in 35:39){
    s1 <- sum(d1[, i], na.rm=TRUE)
    n1 <- sum(!is.na(d1[, i]))
    print(names(d1[i]))
    print(s1/n1)
    print(binom.confint(s1, n1, conf.level = 0.95,
                                methods="bayes")[-1])
    }
###
###
### change in KPS
k1 <- with(d1, lastKps - kpsLmd)
sum(!is.na(k1))
summary(k1)
xtabs( ~ k1 + lastKps, data=d1)
###
### last KPS >= 70
d1$kGr70 <- ifelse(d1$lastKps > 70, 1, 0)
###
### KPS drop of >= 10
d1$k1gr10 <- ifelse(k1>=10, 1, 0)
x1 <- xtabs( ~ k1gr10 + lastKps, data=d1)
library(vcdExtra) # for CMHtest
CMHtest(x1, cscores=as.numeric(dimnames(x1)$lastKps))
library(ryouready) # for Somers D
ord.somers.d(x1)
with(d1, cor.test(k1gr10, lastKps, method="kendall", exact=FALSE))
###
### KPS drop of >= 20
d1$k1gr20 <- ifelse(k1>=20, 1, 0)
(x1 <- xtabs( ~ k1gr20 + lastKps, data=d1))
summary(x1)
CMHtest(x1, cscores=as.numeric(dimnames(x1)$lastKps))
ord.somers.d(x1)
with(d1, cor.test(k1gr20, lastKps, method="kendall", exact=FALSE))
###
### origin of LMD
d1$origLmd
s1 <- sum(grepl("LV", d1$origLmd))
s2 <- sum(!is.na(d1$origLmd))
c(s1/s2, binom.confint(s1, s2, conf.level = 0.95, methods="bayes")[-1])
sum(grepl("cortex", d1$origLmd))
sum(grepl("STS", d1$origLmd))
sum(grepl("TF", d1$origLmd))
```

```
sum(grepl("CS", d1$origLmd))
sum(grepl("\\+", d1$origLmd))
###
### spinal cord imaging
s1 <- sum(d1$wSc, na.rm=TRUE)
s2 <- sum(!is.na(d1$wSc))
c(s1/s2, binom.confint(s1, s2, conf.level = 0.95, methods="bayes")[-1])
### (partial)
s1 <- sum(d1$pSc)
s2 <- sum(!is.na(d1$pSc))
c(s1/s2, binom.confint(s1, s2, conf.level = 0.95, methods="bayes")[-1])
###
### symptoms leading to imaging of whole cord
(t1 <- with(d1[d1$wSc==1, ], table(lmdSympt, wSc)) )
### (check which cases have symptoms attributable to myelopathy)
s2 <- nrow(t1)
c(9/s2, binom.confint(9, s2, conf.level = 0.95, methods="bayes")[-1])
### by symptom
with(d1, table(gait, pSc))
with(d1, table(pain, pSc))
###
### spread
d1$lmdSpread
s1 <- sum(!grepl("none", d1$lmdSpread))
s2 <- sum(!is.na(d1$lmdSpread))
c(s1/s2, binom.confint(s1, s2, conf.level = 0.95, methods="bayes")[-1])
### (cortex)
s1 <- sum(grepl("cortex", d1$lmdSpread))
c(s1/s2, binom.confint(s1, s2, conf.level = 0.95, methods="bayes")[-1])
### (spine)
s1 <- sum(grepl("spine", d1$lmdSpread))
c(s1/s2, binom.confint(s1, s2, conf.level = 0.95, methods="bayes")[-1])
### (cauda)
s1 <- sum(grepl("cauda", d1$lmdSpread))
c(s1/s2, binom.confint(s1, s2, conf.level = 0.95, methods="bayes")[-1])
###
### origin when ends in cortex
with(d1[grepl("cortex", d1$lmdSpread), ], table(origLmd, lmdSpread))
###
### CSF
###
names(d1)[47:53]
### remove summarise from vcdExtra
detach(package:vcdExtra)
library(plyr)
### (from Ommaya)
(t1 <- table(d1$csfOmm))
s1 <- t1[[2]]
s2 <- sum(t1)
c(s1/s2, binom.confint(s1, s2, conf.level = 0.95, methods="bayes")[-1])
### (standard error)
stderr <- function(x) round(sqrt(var(x, na.rm=TRUE) /
                              length(na.omit(x))), 1)
mean1 <- function(x) round(mean(x, na.rm=TRUE), 1)
t(ddply(d1[, 47:53], .(csfOmm), colwise(mean1)))
t(ddply(d1[, 47:53], .(csfOmm), colwise(stderr)))
with(d1, table(csfOmm, csfM0))
with(d1, t(table(csfCyt, csfOmm)))
###
### hospice?
###
```

```
s1 <- sum(d1$hospLmd, na.rm=TRUE)
s2 <- sum(!is.na(d1$hospLmd))
c(s1/s2, binom.confint(s1, s2, conf.level = 0.95, methods="bayes")[-1])
(t1 <- with(d1, table(hospLmd, kpsLmd)))
ord.somers.d(t1)
with(d1, cor.test(hospLmd, kpsLmd, method="kendall", exact=FALSE))
with(d1, cor.test(hospLmd, ageLmd, method="kendall", exact=FALSE))
with(d1, cor.test(hospLmd, lmdGbm, method="kendall", exact=FALSE))
###
### known treatment
###
x1 <- xtabs( ~ it + rt + ct, data=d1[!(d1$hospLmd), ])
(f1 <- ftable(x1))
### remove patient who received no Tx to get total
s2 <- sum(f1) - f1[1,1]
sum(grepl("none", d1$txLmd))
with(d1, t(table(hospLmd, txLmd=="none")))
###
### CT
###
table(d1$ctLmd)
(t1 <- table(d1$ct))
s1 <- t1[[2]]
c(s1/s2, binom.confint(s1, s2, conf.level = 0.95, methods="bayes")[-1])
with(d1, table(ctLmd))
with(d1, xtabs( ~ ctLmd + pathLmd))
s1 <- with(d1, sum(ctLmd=="TMZ", na.rm=TRUE))
c(s1/s2, binom.confint(s1, s2, conf.level = 0.95, methods="bayes")[-1])
###
### i.t. CT
###
(t1 <- table(d1$itTx))
(s1 <- t1[[1]] + t1[[2]] + t1[[4]])
c(s1/s2, binom.confint(s1, s2, conf.level = 0.95, methods="bayes")[-1])
with(d1, table(itTx, mIt))
###
### it and VPS ?
###
with(d1, table(itTx, vps))
###
### VPS
###
(t1 <- table(d1$vps))
xtabs( ~ vps + it, data=d1)
### VPS before Lmd
(t1 <- table(d1$ttVps))
xtabs( ~ vpsBeforeLmd + it, data=d1)
with(d1[d1$vpsBeforeLmd==1, ], table(ttVps - ttLmd))
with(d1[d1$vpsBeforeLmd==1, ], summary(ttVps - ttLmd))
###
### RT
###
(t1 <- table(d1$rt))
s1 <- t1[[2]]
c(s1/s2, binom.confint(s1, s2, conf.level = 0.95, methods="bayes")[-1])
with(d1, table(rtLmd))
with(d1, xtabs( ~ ctLmd + pathLmd))
###
###
###----------------------------------------
### survival - time to progression from LMD
```

```
###---------------------------------------
###
### age
###
(s1 <- survfit(Surv(ttpLmd, e) ~ ageLmd, data=d1))
sig(c1 <- coxph(Surv(ttpLmd, e) ~ ageLmd, data=d1))
###
### gender
###
(s1 <- survfit(Surv(ttpLmd, e) ~ sex, data=d1))
sig(c1 <- coxph(Surv(ttpLmd, e) ~ sex, data=d1))
###
### path
###
(s1 <- survfit(Surv(ttpLmd, e) ~ pathLmd, data=d1))
sig(c1 <- coxph(Surv(ttpLmd, e) ~ pathLmd, data=d1))
### GBM vs others
(s1 <- survfit(Surv(ttpLmd, e) ~ lmdGbm, data=d1))
sig(c1 <- coxph(Surv(ttpLmd, e) ~ lmdGbm, data=d1))
wilcox.test(ttpLmd ~ lmdGbm, data=d1, exact=FALSE)
###
### any Tx effective?
###
d1$anyTxLmd <- d1$it | d1$ct | d1$rt
(s1 <- survfit(Surv(ttpLmd, e) ~ anyTxLmd, data=d1))
sig(c1 <- coxph(Surv(ttpLmd, e) ~ anyTxLmd, data=d1))
wilcox.test(ttpLmd ~ anyTxLmd, data=d1, exact=FALSE)
###
### i.t. CT
###
(s1 <- survfit(Surv(ttpLmd, e) ~ it, data=d1))
sig(c1 <- coxph(Surv(ttpLmd, e) ~ it, data=d1))
wilcox.test(ttpLmd ~ it, data=d1, exact=FALSE)
with(d1, table(it, vps))
###
### systemic CT
###
(s1 <- survfit(Surv(ttpLmd, e) ~ ct, data=d1))
sig(c1 <- coxph(Surv(ttpLmd, e) ~ ct, data=d1))
wilcox.test(ttpLmd ~ ct, data=d1, exact=FALSE)
autoplot(s1)
comp(c1)
###
### any RT
###
(s1 <- survfit(Surv(ttpLmd, e) ~ rt, data=d1))
sig(c1 <- coxph(Surv(ttpLmd, e) ~ rt, data=d1))
wilcox.test(ttpLmd ~ rt, data=d1, exact=FALSE)
###
### it + RT
###
(s1 <- survfit(Surv(ttpLmd, e) ~ itRt, data=d1))
sig(c1 <- coxph(Surv(ttpLmd, e) ~ itRt, data=d1))
sig(c1 <- coxph(Surv(ttpLmd, e) ~ it * rt, data=d1))
with(d1, table(itRt, kpsLmd))
x1 <- xtabs( ~ itRt + kpsLmd, data=d1)
vcdExtra::CMHtest(x1, cscores=as.numeric(dimnames(x1)$kpsLmd))
###
### CT + RT
###
(s1 <- survfit(Surv(ttpLmd, e) ~ ctRt, data=d1))
```

```
sig(c1 <- coxph(Surv(ttpLmd, e) ~ ctRt, data=d1))
wilcox.test(ttpLmd ~ ctRt, data=d1, exact=FALSE)
###
### it + CT
###
(s1 <- survfit(Surv(ttpLmd, e) ~ itCt, data=d1))
sig(c1 <- coxph(Surv(ttpLmd, e) ~ itCt, data=d1))
wilcox.test(ttpLmd ~ itCt, data=d1, exact=FALSE)
###
### it + RT + CT
###
(s1 <- survfit(Surv(ttpLmd, e) ~ itRtCt, data=d1))
sig(c1 <- coxph(Surv(ttpLmd, e) ~ itRtCt, data=d1))
wilcox.test(ttpLmd ~ itRtCt, data=d1, exact=FALSE)
###
### KPS at Dx LM
###
summary(d1$kpsLmd[!(d1$hospLmd)])
(s1 <- survfit(Surv(ttpLmd, e) ~ kpsLmd, data=d1))
sig(c1 <- coxph(Surv(ttpLmd, e) ~ kpsLmd, data=d1))
### hazard ratio for 5-point increase
exp(5*coef(c1))
### ordered factor
summary(c1 <- coxph(Surv(ttpLmd, e) ~ factor(kpsLmd, ordered=TRUE),
                    data=d1))
### check for cutpoint
cutp(c1, var="kpsLmd")
### KPS >= 90
d1$kGr90 <- ifelse(d1$kpsLmd >= 90, 1, 0)
(s1 <- survfit(Surv(ttpLmd, e) ~ kGr90, data=d1))
sig(c1 <- coxph(Surv(ttpLmd, e) ~ kGr90, data=d1))
wilcox.test(ttpLmd ~ kGr90, data=d1, exact=FALSE)
### KPS >= 70 (more meaningful clinically)
d1$kGr70 <- ifelse(d1$kpsLmd >= 70, 1, 0)
(s1 <- survfit(Surv(ttpLmd, e) ~ kGr70, data=d1))
sig(c1 <- coxph(Surv(ttpLmd, e) ~ kGr70, data=d1))
wilcox.test(ttpLmd ~ kGr70, data=d1, exact=FALSE)
###
### last KPS prior to Dx
###
summary(d1$lastKps[!(d1$hospLmd)])
with(d1[!(d1$hospLmd), ], t(table(lastKps, kpsLmd)))
(s1 <- survfit(Surv(ttpLmd, e) ~ lastKps, data=d1))
sig(c1 <- coxph(Surv(ttpLmd, e) ~ lastKps, data=d1))
### hazard ratio for 5-point increase
exp(5*coef(c1))
### ordered factor
summary(c1 <- coxph(Surv(ttpLmd, e) ~ factor(lastKps, ordered=TRUE),
                    data=d1))
###
### spinal mets
###
table(d1$sm)
(t1 <- with(d1, table(sm, lmdGbm)))
summary(t1)
(t1 <- with(d1, table(sm, kpsLmd)))
d1$sm <- as.numeric(d1$sm)
with(d1, cor.test(sm, kpsLmd, method="kendall", exact=FALSE))
###
(s1 <- survfit(Surv(ttpLmd, e) ~ sm, data=d1))
sig(c1 <- coxph(Surv(ttpLmd, e) ~ sm, data=d1))
```

```
summary(c1)
autoplot(s1)
a1 <- autoplot(s1, xlab="Time (months)",
               title="Time to progression by presence of spinal spread of LM",
               survSize=1,
               type="CI", alpha=0.8,
               legLabs=c("No", "Yes"),
               legTitle="Spinal \nspread",
               legTextSize=15,
               pval=TRUE, pX=0.75, pY=0.75)
autoplot(a1)
jpeg(file="ttpSm2.jpg")
autoplot(a1)
dev.off()
###
###----------------------------------------
### time to death/last observation from LMD
###----------------------------------------
###
### age
###
(s1 <- survfit(Surv(ttlLmd, d) ~ ageLmd, data=d1))
sig(c1 <- coxph(Surv(ttlLmd, d) ~ ageLmd, data=d1))
###
### gender
###
(s1 <- survfit(Surv(ttlLmd, d) ~ sex, data=d1))
sig(c1 <- coxph(Surv(ttlLmd, d) ~ sex, data=d1))
###
### path
###
(s1 <- survfit(Surv(ttlLmd, d) ~ pathLmd, data=d1))
sig(c1 <- coxph(Surv(ttlLmd, d) ~ pathLmd, data=d1))
### GBM vs others
(s1 <- survfit(Surv(ttlLmd, d) ~ lmdGbm, data=d1))
sig(c1 <- coxph(Surv(ttlLmd, d) ~ lmdGbm, data=d1))
###
### any Tx effective?
###
(s1 <- survfit(Surv(ttlLmd, d) ~ anyTxLmd, data=d1))
sig(c1 <- coxph(Surv(ttlLmd, d) ~ anyTxLmd, data=d1))
xtabs( ~ kpsLmd + anyTxLmd, data=d1)
wilcox.test(kpsLmd ~ anyTxLmd, data=d1, exact=FALSE)
autoplot(s1)
a1 <- autoplot(s1, xlab="Time (months)",
               title="Time to death by treatment \n (Chemotherapy and/or radiation)",
               survSize=1,
               type="CI", alpha=0.8,
               legLabs=c("No", "Yes"),
               legTitle="Treatment",
               legTextSize=15,
               pval=TRUE, pX=0.75, pY=0.75)
autoplot(a1)
jpeg(file="ttdTx2.jpg")
autoplot(a1)
dev.off()
###
### i.t. CT
###
(s1 <- survfit(Surv(ttlLmd, d) ~ it, data=d1))
sig(c1 <- coxph(Surv(ttlLmd, d) ~ it, data=d1))
```

```
###
### CT
###
(s1 <- survfit(Surv(ttlLmd, d) ~ ct, data=d1))
sig(c1 <- coxph(Surv(ttlLmd, d) ~ ct, data=d1))
###
### RT
###
(s1 <- survfit(Surv(ttlLmd, d) ~ rt, data=d1))
sig(c1 <- coxph(Surv(ttlLmd, d) ~ rt, data=d1))
autoplot(s1)
a1 <- autoplot(s1, xlab="Time (months)",
               title="Time to death by use of radiotherapy",
               survSize=1,
               type="CI", alpha=0.8,
               legLabs=c("No", "Yes"),
               legTitle="Radiotherapy",
               legTextSize=15,
               pval=TRUE, pX=0.75, pY=0.75)
autoplot(a1)
jpeg(file="ttdRT2.jpg")
autoplot(a1)
dev.off()
###
### it + RT
###
(s1 <- survfit(Surv(ttlLmd, d) ~ itRt, data=d1))
sig(c1 <- coxph(Surv(ttlLmd, d) ~ itRt, data=d1))
###
### RT + CT
###
(s1 <- survfit(Surv(ttlLmd, d) ~ ctRt, data=d1))
sig(c1 <- coxph(Surv(ttlLmd, d) ~ ctRt, data=d1))
###
### it + CT
###
(s1 <- survfit(Surv(ttlLmd, d) ~ itCt, data=d1))
sig(c1 <- coxph(Surv(ttlLmd, d) ~ itCt, data=d1))
###
### it + RT + CT
###
(s1 <- survfit(Surv(ttlLmd, d) ~ itRtCt, data=d1))
sig(c1 <- coxph(Surv(ttlLmd, d) ~ itRtCt, data=d1))
###
### KPS at Dx LM
###
(s1 <- survfit(Surv(ttlLmd, d) ~ kpsLmd, data=d1))
sig(c1 <- coxph(Surv(ttlLmd, d) ~ kpsLmd, data=d1))
### hazard ratio for 5-point increase
exp(5*coef(c1))
### ordered factor
summary(c1 <- coxph(Surv(ttlLmd, d) ~ factor(kpsLmd, ordered=TRUE),
                    data=d1))
### check for cutpoint
c1 <- coxph(Surv(ttlLmd, d) ~ kpsLmd, data=d1)
cutp(c1, var="kpsLmd")
### KPS >= 65
d1$kGr65 <- ifelse(d1$kpsLmd >= 65, 1, 0)
(s1 <- survfit(Surv(ttlLmd, d) ~ kGr65, data=d1))
sig(c1 <- coxph(Surv(ttlLmd, d) ~ kGr65, data=d1))
### KPS >= 90
```

```
(s1 <- survfit(Surv(ttlLmd, d) ~ kGr90, data=d1))
sig(c1 <- coxph(Surv(ttlLmd, d) ~ kGr90, data=d1))
autoplot(s1)
a1 <- autoplot(s1, xlab="Time (months)",
               title="Time to death by KPS \n (at time of diagnosis of LM)",
               survSize=1,
               type="CI", alpha=0.8,
               legLabs=c("No", "Yes"),
               legTitle="KPS \n >= 90",
               legTextSize=15,
               pval=TRUE, pX=0.85, pY=0.85)
autoplot(a1)
jpeg(file="ttdKps2.jpg")
autoplot(a1)
dev.off()
###
### last KPS prior to Dx
(s1 <- survfit(Surv(ttlLmd, d) ~ lastKps, data=d1))
sig(c1 <- coxph(Surv(ttlLmd, d) ~ lastKps, data=d1))
### hazard ratio for 5-point increase
exp(5*coef(c1))
### ordered factor
summary(c1 <- coxph(Surv(ttlLmd, d) ~ factor(lastKps, ordered=TRUE),
                    data=d1))
###
### spinal mets
###
(s1 <- survfit(Surv(ttlLmd, d) ~ sm, data=d1))
sig(c1 <- coxph(Surv(ttlLmd, d) ~ sm, data=d1))
(t1 <- with(d1, table(rt, sm)))
summary(t1)
###
###-----------------------------------------
### survival: two-variable models
###-----------------------------------------
###
### ttd: any Tx + KPS
###
xtabs( ~ anyTxLmd + kGr90, data=d1)
ftable(xtabs( ~ anyTxLmd + kGr90 +d, data=d1))
(s1 <- survfit(Surv(ttlLmd, d) ~
               anyTxLmd + kpsLmd, data=d1))
sig(c1 <- coxph(Surv(ttlLmd, d) ~
             anyTxLmd + kpsLmd, data=d1))
(s1 <- survfit(Surv(ttlLmd, d) ~
               anyTxLmd + kGr90, data=d1))
sig(c1 <- coxph(Surv(ttlLmd, d) ~
             anyTxLmd + kGr90, data=d1))
summary(c1)
comp(c1)
autoplot(s1, pval=TRUE)
a1 <- autoplot(s1, xlab="Time (months)",
               title="Time to death by KPS and use of any treatment",
               survSize=1,
               type="CI", alpha=0.8,
               legLabs=c("No Tx\nKPS<90",
               "Some Tx\nKPS<90", "Some Tx\nKPS>=90"),
               legTextSize=15,
               legSize=4)
a1$plot <- a1$plot +
    annotate("text", x=18, y=0.95,
```

```
              label="Log-rank\ntest\np = 0.06") +
     annotate("text", x=3, y=0.15,
              label="Log-rank\ntest\nfor trend\np = 0.99")
autoplot(a1, plotHeight=0.7, tabHeight=0.3)
jpeg(file="ttdKpsTx2.jpg")
autoplot(a1, plotHeight=0.7, tabHeight=0.3)
dev.off()
###
### ttd: RT + KPS
###
ftable(xtabs( ~ rt + kGr90 +d, data=d1))
(s1 <- survfit(Surv(ttlLmd, d) ~
                rt + kpsLmd, data=d1))
sig(c1 <- coxph(Surv(ttlLmd, d) ~
              rt + kpsLmd, data=d1))
(s1 <- survfit(Surv(ttlLmd, d) ~
                rt + kGr90, data=d1))
sig(c1 <- coxph(Surv(ttlLmd, d) ~
              rt + kGr90, data=d1))
summary(c1)
comp(c1)
autoplot(s1)
###
###----------------------------------------
### Tx after initial progression
###----------------------------------------
###
(t1 <- table(d1$anyTxProg))
s1 <- t1[[2]]
s2 <- sum(t1)
c(s1/s2, binom.confint(s1, s2, conf.level = 0.95, methods="bayes")[-1])
### survival
(survfit(Surv(ttlLmd, d) ~ 1, data=d1[d1$anyTxProg==1, ]) )
(t2 <- table(d1$noProgLmd))
s1 <- t2[[2]]
s2 <- sum(t2) - t1[[1]]
c(s1/s2, binom.confint(s1, s2, conf.level = 0.95, methods="bayes")[-1])
xtabs( ~ noProgLmd + pathLmd, data=d1)
###
###
###----------------------------------------
### discussion
###----------------------------------------
###
###
### percentages for table
###
100 * sum(d1$lmdGbm, na.rm=TRUE) / length(na.omit(d1$lmdGbm))
100 * sum(d1$ct, na.rm=TRUE) / length(na.omit(d1$ct))
100 * sum(d1$rt, na.rm=TRUE) / length(na.omit(d1$rt))
100 * sum(d1$it, na.rm=TRUE) / length(na.omit(d1$it))
###
### overall median ttp and ttd
###
(s1 <- survfit(Surv(ttpLmd, e) ~ 1, data=d1))
(s1 <- survfit(Surv(ttlLmd, d) ~ 1, data=d1))
###
### spinal mets with GBM
###
sum(d1$lmdGbm==1 & d1$sm)
(s1 <- survfit(Surv(ttlLmd, d) ~ sm,
```

```
                data=d1[d1$lmdGbm==1, ]))
###
### spinal mets all causes
###
(s1 <- survfit(Surv(ttpLmd, e) ~ sm, data=d1))
(s1 <- survfit(Surv(ttlLmd, d) ~ sm, data=d1))
###
### use of CT, RT in our series
###
xtabs( ~ sm + ct, data=d1)
xtabs( ~ sm + rt, data=d1)
xtabs( ~ sm + it, data=d1)
###
### Tx with TMZ and RT
###
(s1 <- survfit(Surv(ttpLmd, e) ~ 1,
                data=d1[d1$rt==1 & grepl("TMZ", d1$ctLmd), ]))
(s1 <- survfit(Surv(ttlLmd, d) ~ 1,
                data=d1[d1$rt==1 & grepl("TMZ", d1$ctLmd), ]))
###
### incidence
100 * sum(grepl("GBM", d1$pathDx)) / (10 * 60)
###
### spinal cord involvement: ttp & ttd
survfit(Surv(ttpLmd, e) ~ 1, data=d1[grepl("spine", d1$lmdSpread), ])
survfit(Surv(ttlLmd, d) ~ 1, data=d1[grepl("spine", d1$lmdSpread), ])
###
### shunt by no. surgeries
(t1 <- with(d1, table(vps, noSxProg>=1)))
fisher.test(t1)
###
### 4th ventricle involved
sum(grepl("4V|4th", d1$origLmd)) +
    sum(grepl("4V|4th", d1$lmdSpread))
table(grepl("4V|4th", d1$origLmd) |
      grepl("4V|4th", d1$lmdSpread),
      d1$naus)
###
### Depocyt
sum(grepl("DC", d1$itTx))
survfit(Surv(ttpLmd, e) ~ 1, data=d1[grepl("DC", d1$itTx), ])
table(grepl("DC", d1$itTx),
      d1$vps)
###
###----------------------------------------
### sub-analysis, GBM only
###----------------------------------------
###
d2 <- d1[d1$lmdGbm==1, ]
nrow(d2)
###
###----------------------------------------
### survival - time to progression from LMD
###----------------------------------------
###
### age
###
table(d2$ageLmd)
(s1 <- survfit(Surv(ttpLmd, e) ~ ageLmd, data=d2))
sig(c1 <- coxph(Surv(ttpLmd, e) ~ ageLmd, data=d2))
###
```

```
### gender
###
table(d2$sex)
(s1 <- survfit(Surv(ttpLmd, e) ~ sex, data=d2))
sig(c1 <- coxph(Surv(ttpLmd, e) ~ sex, data=d2))
###
### any Tx effective?
###
table(d2$anyTxLmd)
(s1 <- survfit(Surv(ttpLmd, e) ~ anyTxLmd, data=d2))
sig(c1 <- coxph(Surv(ttpLmd, e) ~ anyTxLmd, data=d2))
###
### i.t. CT
###
table(d2$it)
(s1 <- survfit(Surv(ttpLmd, e) ~ it, data=d2))
sig(c1 <- coxph(Surv(ttpLmd, e) ~ it, data=d2))
###
### systemic CT
###
table(d2$ct)
(s1 <- survfit(Surv(ttpLmd, e) ~ ct, data=d2))
sig(c1 <- coxph(Surv(ttpLmd, e) ~ ct, data=d2))
###
### any RT
###
table(d2$rt)
(s1 <- survfit(Surv(ttpLmd, e) ~ rt, data=d2))
sig(c1 <- coxph(Surv(ttpLmd, e) ~ rt, data=d2))
###
### it + RT - counterintuitive
###
table(d2$itRt)
(s1 <- survfit(Surv(ttpLmd, e) ~ itRt, data=d2))
sig(c1 <- coxph(Surv(ttpLmd, e) ~ itRt, data=d2))
###
### CT + RT
###
table(d2$ctRt)
(s1 <- survfit(Surv(ttpLmd, e) ~ ctRt, data=d2))
sig(c1 <- coxph(Surv(ttpLmd, e) ~ ctRt, data=d2))
###
### it + CT
###
table(d2$itCt)
(s1 <- survfit(Surv(ttpLmd, e) ~ itCt, data=d2))
sig(c1 <- coxph(Surv(ttpLmd, e) ~ itCt, data=d2))
###
### it + RT + CT - counterintuitive
###
table(d2$itRtCt)
(s1 <- survfit(Surv(ttpLmd, e) ~ itRtCt, data=d2))
sig(c1 <- coxph(Surv(ttpLmd, e) ~ itRtCt, data=d2))
###
### KPS at Dx LM
###
table(d2$kpsLmd)
(s1 <- survfit(Surv(ttpLmd, e) ~ kpsLmd, data=d2))
sig(c1 <- coxph(Surv(ttpLmd, e) ~ kpsLmd, data=d2))
summary(c1 <- coxph(Surv(ttpLmd, e) ~
                factor(kpsLmd, ordered=TRUE),
```

```
                data=d2))
###
### last KPS prior to Dx
###
table(d2$lastKps)
(s1 <- survfit(Surv(ttpLmd, e) ~ lastKps, data=d2))
sig(c1 <- coxph(Surv(ttpLmd, e) ~ lastKps, data=d2))
###
### spinal mets
###
table(d2$sm)
(s1 <- survfit(Surv(ttpLmd, e) ~ sm, data=d2))
sig(c1 <- coxph(Surv(ttpLmd, e) ~ sm, data=d2))
###
###----------------------------------------
### time to death/last observation from LMD
###----------------------------------------
###
### age
###
(s1 <- survfit(Surv(ttlLmd, d) ~ ageLmd, data=d2))
sig(c1 <- coxph(Surv(ttlLmd, d) ~ ageLmd, data=d2))
###
### gender
###
(s1 <- survfit(Surv(ttlLmd, d) ~ sex, data=d2))
sig(c1 <- coxph(Surv(ttlLmd, d) ~ sex, data=d2))
###
### any Tx effective?
###
(s1 <- survfit(Surv(ttlLmd, d) ~ anyTxLmd, data=d2))
sig(c1 <- coxph(Surv(ttlLmd, d) ~ anyTxLmd, data=d2))
### due to KPS?
xtabs( ~ + kpsLmd + anyTxLmd, data=d2)
wilcox.test(kpsLmd ~ anyTxLmd, data=d2, exact=FALSE)
###
### i.t. CT
###
(s1 <- survfit(Surv(ttlLmd, d) ~ it, data=d2))
sig(c1 <- coxph(Surv(ttlLmd, d) ~ it, data=d2))
###
### CT
###
(s1 <- survfit(Surv(ttlLmd, d) ~ ct, data=d2))
sig(c1 <- coxph(Surv(ttlLmd, d) ~ ct, data=d2))
###
### RT
###
(s1 <- survfit(Surv(ttlLmd, d) ~ rt, data=d2))
sig(c1 <- coxph(Surv(ttlLmd, d) ~ rt, data=d2))
xtabs( ~ + kpsLmd + rt, data=d2)
wilcox.test(kpsLmd ~ rt, data=d2, exact=FALSE)
###
### it + RT
###
(s1 <- survfit(Surv(ttlLmd, d) ~ itRt, data=d2))
sig(c1 <- coxph(Surv(ttlLmd, d) ~ itRt, data=d2))
###
### RT + CT
###
(s1 <- survfit(Surv(ttlLmd, d) ~ ctRt, data=d2))
```

```
sig(c1 <- coxph(Surv(ttlLmd, d) ~ ctRt, data=d2))
###
### it + CT
###
(s1 <- survfit(Surv(ttlLmd, d) ~ itCt, data=d2))
sig(c1 <- coxph(Surv(ttlLmd, d) ~ itCt, data=d2))
###
### it + RT + CT
###
(s1 <- survfit(Surv(ttlLmd, d) ~ itRtCt, data=d2))
sig(c1 <- coxph(Surv(ttlLmd, d) ~ itRtCt, data=d2))
###
### KPS at Dx LM
###
(s1 <- survfit(Surv(ttlLmd, d) ~ kpsLmd, data=d2))
sig(c1 <- coxph(Surv(ttlLmd, d) ~ kpsLmd, data=d2))
### hazard ratio for 5-point increase
exp(5*coef(c1))
### ordered factor
summary(c1 <- coxph(Surv(ttlLmd, d) ~ factor(kpsLmd, ordered=TRUE),
                    data=d2))
###
### last KPS prior to Dx
(s1 <- survfit(Surv(ttlLmd, d) ~ lastKps, data=d2))
sig(c1 <- coxph(Surv(ttlLmd, d) ~ lastKps, data=d2))
### hazard ratio for 5-point increase
exp(5*coef(c1))
### ordered factor
summary(c1 <- coxph(Surv(ttlLmd, d) ~ factor(lastKps, ordered=TRUE),
                    data=d2))
###
### spinal mets
###
(s1 <- survfit(Surv(ttlLmd, d) ~ sm, data=d2))
sig(c1 <- coxph(Surv(ttlLmd, d) ~ sm, data=d2))
(t1 <- with(d2, table(rt, sm)))
###
###----------------------------------------
### survival: two-variable models
###----------------------------------------
###
### ttd: anyTx + KPS
###
(s1 <- survfit(Surv(ttlLmd, d) ~
               anyTxLmd + kpsLmd, data=d2))
sig(c1 <- coxph(Surv(ttlLmd, d) ~
             anyTxLmd + kpsLmd, data=d2))
summary(c1 <- coxph(Surv(ttlLmd, d) ~
             anyTxLmd + factor(kpsLmd, ordered=TRUE), data=d2))
(s1 <- survfit(Surv(ttlLmd, d) ~
               anyTxLmd + kGr90, data=d2))
sig(c1 <- coxph(Surv(ttlLmd, d) ~
             anyTxLmd + kGr90, data=d2))
summary(c1)
###
### ttd: RT + KPS
###
ftable(xtabs( ~ rt + kGr90 +d, data=d2))
(s1 <- survfit(Surv(ttlLmd, d) ~
               rt + kpsLmd, data=d2))
sig(c1 <- coxph(Surv(ttlLmd, d) ~
```

```
              rt + kpsLmd, data=d2))
summary(c1)
(s1 <- survfit(Surv(ttlLmd, d) ~
                rt + kGr90, data=d2))
sig(c1 <- coxph(Surv(ttlLmd, d) ~
              rt + kGr90, data=d2))
###
###----------------------------------------
###----------------------------------------
### original 41-case series
###----------------------------------------
###----------------------------------------
###
###----------------------------------------
### abstract
###----------------------------------------
###
sum(d1$lmdAtDx)
sum(d1$lmdFirstProg, na.rm=TRUE)
###
### all experienced progression:
### (for model fitting, NA's in predictor
### variables will exclude cases as required)
d1$e <- rep(1, nrow(d1))
###
### no. died
sum(d1$d, na.rm=TRUE)
### time to death
summary(d1$ttlLmd[d1$d==1])
###
### overall median ttp and ttd
###
(s1 <- survfit(Surv(ttpLmd, e) ~ 1, data=d1))
summary(d1$ttpLmd)
sum(d1$d, na.rm=TRUE)
(s1 <- survfit(Surv(ttlLmd, d) ~ 1, data=d1))
summary(d1$ttlLmd[d1$d==1])
###
### any Tx effective?
###
### ttp
d1$anyTxLmd <- d1$it | d1$ct | d1$rt
(s1 <- survfit(Surv(ttpLmd, e) ~ anyTxLmd, data=d1))
(c1 <- coxph(Surv(ttpLmd, e) ~ anyTxLmd, data=d1))
sig(c1)
wilcox.test(ttpLmd ~ anyTxLmd, data=d1, exact=FALSE)
### ttd
(c1 <- coxph(Surv(ttlLmd, d) ~ anyTxLmd, data=d1))
sig(c1)
###
### on i.t.Tx with shunt
with(d1, table(it, vps))
###
###----------------------------------------
### descriptives at time of Dx
###----------------------------------------
###
### latex table
library(xtable)
###
### this one needs landscape format
```

```
###
d2 <- as.matrix(d1[, c("ageLmd", "sex", "ttLmd", "pathLmd",
                       "lmdSpread", "txLmd",
                       "ttpLmd", "ttlLmd", "d"
                       )])
rownames(d2) <- rep("", nrow(d2))
colnames(d2) <- c("Age", "Sex", "ttLmd", "Path",
                  "Location (of spread)",
                  "Tx", "ttP", "TTD", "D")
x1 <- xtable(d2)
print(x1, include.rownames = FALSE, NA.string = "NA")
###
### standard table format
###
d2 <- as.matrix(d1[, c("ageLmd", "sex", "ttLmd", "pathLmd",
                       "txLmd",
                       "ttpLmd", "ttlLmd", "d"
                       )])
colnames(d2) <- c("Age", "Sex", "ttLmd", "Path",
                  "Tx", "ttP", "ttD", "D")
d2 <- as.data.frame(d2)
d2$gr <- NA
d2$gr[d2$Path=="GBM"] <- 4
d2$gr[d2$Path=="AA" | d2$Path=="AOA" | d2$Path=="AOD" | d2$Path=="AG"] <- 3
d2$gr[d2$Path=="EP" | d2$Path=="ME" | d2$Path=="OA"] <- 2
levels(d2$Path)[4] <- "AO"
d2 <- d2[order(-d2$gr, d2$ttD), ]
d2 <- d2[-ncol(d2) ]
x1 <- xtable(d2)
print(x1, include.rownames = FALSE, NA.string = "NA")
###
### age
###
summary(d1$ageLmd)
summary(d1$ageLmd[!d1$ageLmd==3])
###
### pathology at Dx
###
table(d1$pathDx)
### GBM at Dx
s1 <- sum(d1$lmdGbm, na.rm=TRUE)
s2 <- sum(is.finite(d1$lmdGbm))
c(s1/s2, binom.confint(s1, s2, conf.level = 0.95, methods="bayes")[-1])
###
### Lmd at Dx
###
s1 <- sum(d1$lmdAtDx, na.rm=TRUE)
s2 <- sum(is.finite(d1$lmdAtDx))
c(s1/s2, binom.confint(s1, s2, conf.level = 0.95, methods="bayes")[-1])
### time to Lmd if not present at Dx
summary(d1$ttLmd[!d1$lmdAtDx==1])
### Lmd not present at time Dx
d2 <- d1[!d1$lmdAtDx, ]
### time to Lmd
summary(d2$ttLmd)
sort(d2$ttLmd)
###
### time to Lmd >5y
###
addmargins(table(d2$ttLmd[d2$ttLmd>60], d2$pathLmd[d2$ttLmd>60]))
d3 <- d2[d2$ttLmd>60, ]
```

```
d4 <- as.matrix(d3[, c("ageDx", "sex", "loc", "pathDx",
                       "noProg", "ttLmd"
                       )])
rownames(d4) <- rep("", nrow(d4))
colnames(d4) <- c("Age", "Sex", "Location",
                  "Path", "Progressions", "ttLMD")
d4 <- as.data.frame(d4)
d4 <- d4[order(d4$Path, d4$ttLMD), ]
x1 <- xtable(d4)
print(x1, include.rownames = FALSE, NA.string = "NA")
###
### Lmd present at first progression
###
s1 <- sum(d1$lmdFirstProg, na.rm=TRUE)
### total Lmd not present initially
s2 <- sum(is.finite(d1$lmdFirstProg))
c(s1/s2, binom.confint(s1, s2, conf.level = 0.95, methods="bayes")[-1])
###
### path~ progressed?
###
s1 <- sum(d1$pathProg, na.rm=TRUE)
s2 <- nrow(d1) - sum(is.na(d1$lmdFirstProg))
c(s1/s2, binom.confint(s1, s2, conf.level = 0.95, methods="bayes")[-1])
xtabs( ~ pathDx + pathLmd, data=d1[d1$pathProg==1, ])
###
###----------------------------------------
### survival analysis - time to LMD
###----------------------------------------
###
### overall (if not present at time of Dx)
(s1 <- survfit(Surv(ttLmd, e) ~ 1, data=d1[!d1$lmdAtDx, ]))
with(d1, summary(ttLmd[!lmdAtDx]))
###
### age
###
(s1 <- survfit(Surv(ttLmd, e) ~ ageDx, data=d1))
(c1 <- coxph(Surv(ttLmd, e) ~ ageDx, data=d1))
### hazard ratio for 10-year increase
exp(10*coef(c1))
### check for cutpoint
cutp(c1, var="ageDx")
d1$ageGr19 <- ifelse(d1$ageDx>19, 0, 1)
(s1 <- survfit(Surv(ttLmd, e) ~ ageGr19, data=d1))
(c1 <- coxph(Surv(ttLmd, e) ~ ageGr19, data=d1))
sig(c1)
###
### gender
###
xtabs(ttLmd ~ sex, data=d1)
(s1 <- coxph(Surv(ttLmd, e) ~ sex, data=d1))
(c1 <- coxph(Surv(ttLmd, e) ~ sex, data=d1))
wilcox.test(ttLmd ~ sex, data=d1)
###
### laterality
###
(s1 <- survfit(Surv(ttLmd, e) ~ lat, data=d1))
(c1 <- coxph(Surv(ttLmd, e) ~ lat, data=d1))
### bilateral vs unilateral
d1$bil <- ifelse(d1$lat=="b", 1, 0)
(s1 <- survfit(Surv(ttLmd, e) ~ bil, data=d1))
(c1 <- coxph(Surv(ttLmd, e) ~ bil, data=d1)
```

```
###
### location
###
(s1 <- survfit(Surv(ttLmd, e) ~ loc, data=d1)
(s1 <- survfit(Surv(ttLmd, e) ~ sLoc, data=d1))
(c1 <- coxph(Surv(ttLmd, e) ~ sLoc, data=d1))
### lobar vs basal ganglia
d1$lb <- d1$sLoc
d1$lb[!d1$sLoc=="l" & !d1$sLoc=="b"] <- NA
(s1 <- survfit(Surv(ttLmd, e) ~ lb, data=d1))
(c1 <- coxph(Surv(ttLmd, e) ~ lb, data=d1))
### increase in risk if basal ganglia
1/exp(coef(c1))
sig(c1)
wilcox.test(ttLmd ~ lb, data=d1, exact=FALSE)
###
### path~
###
(s1 <- survfit(Surv(ttLmd, e) ~ pathDx, data=d1))
(c1 <- coxph(Surv(ttLmd, e) ~ pathDx, data=d1))
### cases with >1 observation
d2 <- d1[d1$pathDx=="AOA"|
         d1$pathDx=="GBM"|
         d1$pathDx=="AOD"|d1$pathDx=="OD", ]
d2 <- transform(d2, pathDx = droplevels(as.factor(pathDx)))
(s1 <- survfit(Surv(ttLmd, rep(1, nrow(d2))) ~
               pathDx, data=d2))
(c1 <- coxph(Surv(ttLmd, rep(1, nrow(d2))) ~
             pathDx, data=d2))
###
### grade
###
(s1 <- survfit(Surv(ttLmd, e) ~ gradeDx, data=d1))
(c1 <- coxph(Surv(ttLmd, e) ~
             as.factor(gradeDx), data=d1))
### GBM vs others
d1$GBM <- d1$gradeDx==4
(s1 <- survfit(Surv(ttLmd, e) ~ GBM, data=d1))
(c1 <- coxph(Surv(ttLmd, e) ~ GBM, data=d1))
wilcox.test(ttLmd ~ GBM, data=d1)
###
### histology
###
names(d1)[11:17]
for (i in 11:17) {
    s1 <- survfit(Surv(ttLmd, e) ~ d1[, i], data=d1)
    c1 <- coxph(Surv(ttLmd, e) ~ d1[, i], data=d1)
    print(names(d1[i]))
    print(s1)
    print(c1)
}
###
### 1p19q
###
xtabs( ~ X1p19q + pathDx, data=d1)
(s1 <- survfit(Surv(ttLmd, e) ~ X1p19q, data=d1))
(c1 <- coxph(Surv(ttLmd, e) ~ X1p19q, data=d1))
sig(c1)
wilcox.test(ttLmd ~ X1p19q, data=d1)
###
### surgery
```

```
###
(s1 <- survfit(Surv(ttLmd, e) ~ sxDx, data=d1))
(c1 <- coxph(Surv(ttLmd, e)~ sxDx, data=d1))
### GTR vs STR
d1$sxDx[!d1$sxDx=="GTR" & !d1$sxDx=="STR"] <- NA
(s1 <- survfit(Surv(ttLmd, e) ~ sxDx, data=d1))
(c1 <- coxph(Surv(ttLmd, e)~ sxDx, data=d1))
wilcox.test(ttLmd ~ sxDx, data=d1, exact=FALSE)
###
### any Tx at diagnosis
###
with(d1, table(sRtDx))
with(d1, table(ctDx))
d1$anyTxDx <-d1$sRtDx!="none" | d1$ctDx!="none"
(s1 <- survfit(Surv(ttLmd, e) ~ anyTxDx, data=d1))
(c1 <- coxph(Surv(ttLmd, e)~ anyTxDx, data=d1))
sig(c1)
###
### RT
###
(s1 <- survfit(Surv(ttLmd, e) ~ sRtDx, data=d1))
(c1 <- coxph(Surv(ttLmd, e)~ sRtDx, data=d1))
### 60t vs none
d1$n60 <- d1$sRtDx
d1$n60[!d1$sRtDx=="60t" & !d1$sRtDx=="none"] <- NA
(s1 <- survfit(Surv(ttLmd, e) ~ n60, data=d1))
(c1 <- coxph(Surv(ttLmd, e)~ n60, data=d1))
### compare RTt and RTtm
d2 <- d1[d1$sRtDx=="60t"|d1$sRtDx=="60tm", ]
d2 <- transform(d2, sRtDx = droplevels(as.factor(sRtDx)))
(s1 <- survfit(Surv(ttLmd, rep(1, nrow(d2))) ~
               sRtDx, data=d2))
(c1 <- coxph(Surv(ttLmd, rep(1, nrow(d2))) ~
            sRtDx, data=d2))
wilcox.test(ttLmd ~ sRtDx, data=d2, exact=FALSE)
###
### CT
###
(s1 <- survfit(Surv(ttLmd, e) ~ ctDx, data=d1))
### TMZ vs none
d2 <- d1[d1$ctDx=="TMZ"|d1$ctDx=="none", ]
(s1 <- survfit(Surv(ttLmd, rep(1, nrow(d2))) ~
               ctDx, data=d2))
(c1 <- coxph(Surv(ttLmd, rep(1, nrow(d2))) ~
             ctDx, data=d2))
wilcox.test(ttLmd ~ ctDx, data=d2, exact=FALSE)
###
### duration of CT
(s1 <- survfit(Surv(ttLmd, e) ~ mCtDx, data=d1))
(c1 <- coxph(Surv(ttLmd, e) ~ mCtDx, data=d1))
###
### no. progressions
###
(s1 <- survfit(Surv(ttLmd, e)~ noProg, data=d1))
(c1 <- coxph(Surv(ttLmd, e)~ noProg, data=d1))
###
###----------------------------------------
### descriptives at Dx LMD
###----------------------------------------
###
### symptoms
```

```
names(d1)[35:39]
for (i in 35:39){
    s1 <- sum(d1[, i], na.rm=TRUE)
    n1 <- sum(!is.na(d1[, i]))
    print(names(d1[i]))
    print(s1/n1)
    print(binom.confint(s1, n1, conf.level = 0.95,
                              methods="bayes")[-1])
    }
###
### change in KPS
###
k1 <- with(d1, lastKps - kpsLmd)
sum(!is.na(k1))
summary(k1)
xtabs( ~ k1 + lastKps, data=d1)
### last KPS >= 70
d1$kGr70 <- ifelse(d1$lastKps > 70, 1, 0)
### KPS drop of >= 10
d1$k1gr10 <- ifelse(k1>=10, 1, 0)
x1 <- xtabs( ~ k1gr10 + lastKps, data=d1)
CMHtest(x1, cscores=as.numeric(dimnames(x1)$lastKps))
ord.somers.d(x1)
### KPS drop of >= 20
d1$k1gr20 <- ifelse(k1>=20, 1, 0)
(x1 <- xtabs( ~ k1gr20 + lastKps, data=d1))
summary(x1)
library(vcdExtra) # for CMHtest
CMHtest(x1, cscores=as.numeric(dimnames(x1)$lastKps))
library(ryouready) # for Somers D
ord.somers.d(x1)
###
### origin of LMD
###
d1$origLmd
s1 <- sum(grepl("LV", d1$origLmd))
s2 <- sum(!is.na(d1$origLmd))
c(s1/s2, binom.confint(s1, s2, conf.level = 0.95, methods="bayes")[-1])
sum(grepl("cortex", d1$origLmd))
sum(grepl("STS", d1$origLmd))
sum(grepl("TF", d1$origLmd))
sum(grepl("CS", d1$origLmd))
sum(grepl("\\+", d1$origLmd))
###
### spinal cord imaging
###
s1 <- sum(d1$wSc, na.rm=TRUE)
s2 <- sum(!is.na(d1$wSc))
c(s1/s2, binom.confint(s1, s2, conf.level = 0.95, methods="bayes")[-1])
### (partial)
s1 <- sum(d1$pSc)
s2 <- sum(!is.na(d1$pSc))
c(s1/s2, binom.confint(s1, s2, conf.level = 0.95, methods="bayes")[-1])
### symptoms leading to imaging of whole cord
(t1 <- with(d1[d1$wSc==1, ], table(lmdSympt, wSc)) )
### (check which cases have symptoms attributable to myelopathy)
s2 <- nrow(t1)
c(9/s2, binom.confint(9, s2, conf.level = 0.95, methods="bayes")[-1])
### by symptom
with(d1, table(gait, pSc))
with(d1, table(pain, pSc))
```

```
###
### spread
###
d1$lmdSpread
s1 <- sum(!grepl("none", d1$lmdSpread))
s2 <- sum(!is.na(d1$lmdSpread))
c(s1/s2, binom.confint(s1, s2, conf.level = 0.95, methods="bayes")[-1])
### (cortex)
s1 <- sum(grepl("cortex", d1$lmdSpread))
c(s1/s2, binom.confint(s1, s2, conf.level = 0.95, methods="bayes")[-1])
### (spine)
s1 <- sum(grepl("spine", d1$lmdSpread))
c(s1/s2, binom.confint(s1, s2, conf.level = 0.95, methods="bayes")[-1])
### (cauda)
s1 <- sum(grepl("cauda", d1$lmdSpread))
c(s1/s2, binom.confint(s1, s2, conf.level = 0.95, methods="bayes")[-1])
###
### origin when ends in cortex
with(d1[grepl("cortex", d1$lmdSpread), ], table(origLmd, lmdSpread))
###
### CSF
###
names(d1)[47:53]
### remove summarize from package:vcdExtra
detach(package:vcdExtra)
library(plyr)
### (from Ommaya)
(t1 <- table(d1$csfOmm))
s1 <- t1[[2]]
s2 <- sum(t1)
c(s1/s2, binom.confint(s1, s2, conf.level = 0.95, methods="bayes")[-1])
### (standard error)
stderr <- function(x) round(sqrt(var(x, na.rm=TRUE) /
                                  length(na.omit(x))), 1)
mean1 <- function(x) round(mean(x, na.rm=TRUE), 1)
t(ddply(d1[, 47:53], .(csfOmm), colwise(mean1)))
t(ddply(d1[, 47:53], .(csfOmm), colwise(stderr)))
with(d1, table(csfOmm, csfM0))
with(d1, table(csfCyt, csfOmm))
###
### hospice?
###
s1 <- sum(d1$hospLmd, na.rm=TRUE)
s2 <- sum(!is.na(d1$hospLmd))
c(s1/s2, binom.confint(s1, s2, conf.level = 0.95, methods="bayes")[-1])
(t1 <- with(d1, table(hospLmd, kpsLmd)))
ord.somers.d(t1)
###
### known treatment
###
x1 <- xtabs( ~ it + rt + ct, data=d1[!(d1$hospLmd), ])
(f1 <- ftable(x1))
s2 <- sum(f1)
sum(grepl("none", d1$txLmd))
with(d1, table(hospLmd, txLmd=="none"))
###
### i.t. CT
###
(t1 <- table(d1$itTx))
(s1 <- t1[[1]] + t1[[2]] + t1[[4]])
c(s1/s2, binom.confint(s1, s2, conf.level = 0.95, methods="bayes")[-1])
```

```
###
### it and VPS ?
###
with(d1, table(itTx, vps))
with(d1, table(itTx, mIt))
###
### CT
###
table(d1$ctLmd)
(t1 <- table(d1$ct))
s1 <- t1[[2]]
c(s1/s2, binom.confint(s1, s2, conf.level = 0.95, methods="bayes")[-1])
with(d1, table(ctLmd))
with(d1, xtabs( ~ ctLmd + pathLmd))
s1 <- with(d1, sum(ctLmd=="TMZ", na.rm=TRUE))
c(s1/s2, binom.confint(s1, s2, conf.level = 0.95, methods="bayes")[-1])
###
### RT
###
(t1 <- table(d1$rt))
s1 <- t1[[2]]
c(s1/s2, binom.confint(s1, s2, conf.level = 0.95, methods="bayes")[-1])
with(d1, table(rtLmd))
with(d1, xtabs( ~ ctLmd + pathLmd))
###
### VPS
###
(t1 <- table(d1$vps))
xtabs( ~ vps + it, data=d1)
xtabs( ~ vps + it, data=d1)
### VPS before Lmd
(t1 <- table(d1$ttVps))
xtabs( ~ vpsBeforeLmd + it, data=d1)
with(d1[d1$vpsBeforeLmd==1, ], table(ttVps - ttLmd) )
###
###
###----------------------------------------
### survival - time to progression from LMD
###----------------------------------------
###
### age
###
(s1 <- survfit(Surv(ttpLmd, e) ~ ageLmd, data=d1))
(c1 <- coxph(Surv(ttpLmd, e) ~ ageLmd, data=d1))
###
### gender
###
(s1 <- survfit(Surv(ttpLmd, e) ~ sex, data=d1))
(c1 <- coxph(Surv(ttpLmd, e) ~ sex, data=d1))
###
### path
###
(s1 <- survfit(Surv(ttpLmd, e) ~ pathLmd, data=d1))
(c1 <- coxph(Surv(ttpLmd, e) ~ pathLmd, data=d1))
### GBM vs others
(s1 <- survfit(Surv(ttpLmd, e) ~ lmdGbm, data=d1))
(c1 <- coxph(Surv(ttpLmd, e) ~ lmdGbm, data=d1))
sig(c1)
wilcox.test(ttpLmd ~ lmdGbm, data=d1, exact=FALSE)
###
### any Tx effective?
```

```
###
d1$anyTxLmd <- d1$it | d1$ct | d1$rt
(s1 <- survfit(Surv(ttpLmd, e) ~ anyTxLmd, data=d1))
(c1 <- coxph(Surv(ttpLmd, e) ~ anyTxLmd, data=d1))
sig(c1)
wilcox.test(ttpLmd ~ anyTxLmd, data=d1, exact=FALSE)
###
### i.t. CT
###
(s1 <- survfit(Surv(ttpLmd, e) ~ it, data=d1))
(c1 <- coxph(Surv(ttpLmd, e) ~ it, data=d1))
sig(c1)
wilcox.test(ttpLmd ~ it, data=d1, exact=FALSE)
with(d1, table(it, vps))
###
### systemic CT
###
(s1 <- survfit(Surv(ttpLmd, e) ~ ct, data=d1))
(c1 <- coxph(Surv(ttpLmd, e) ~ ct, data=d1))
sig(c1)
wilcox.test(ttpLmd ~ ct, data=d1, exact=FALSE)
autoplot(s1)
a1 <- autoplot(s1, xlab="Time (months)",
               title="Time to progression \n by use of systemic chemotherapy",
               survSize=1,
               type="CI", alpha=0.8,
               legLabs=c("No", "Yes"),
               legTitle="Systemic \nchemotherapy",
               legTextSize=15,
               pval=TRUE, pX=0.75, pY=0.75)
autoplot(a1)
jpeg(file="ttpCT")
autoplot(a1)
dev.off()
###
### any RT
###
(s1 <- survfit(Surv(ttpLmd, e) ~ rt, data=d1))
(c1 <- coxph(Surv(ttpLmd, e) ~ rt, data=d1))
wilcox.test(ttpLmd ~ rt, data=d1, exact=FALSE)
###
### it + RT
###
(s1 <- survfit(Surv(ttpLmd, e) ~ itRt, data=d1))
(c1 <- coxph(Surv(ttpLmd, e) ~ itRt, data=d1))
(c1 <- coxph(Surv(ttpLmd, e) ~ it * rt, data=d1))
with(d1, table(itRt, kpsLmd))
x1 <- xtabs( ~ itRt + kpsLmd, data=d1)
vcdExtra::CMHtest(x1, cscores=as.numeric(dimnames(x1)$kpsLmd))
ord.somers.d(x1)
###
### CT + RT
###
(s1 <- survfit(Surv(ttpLmd, e) ~ ctRt, data=d1))
(c1 <- coxph(Surv(ttpLmd, e) ~ ctRt, data=d1))
wilcox.test(ttpLmd ~ ctRt, data=d1, exact=FALSE)
###
### it + CT
###
(s1 <- survfit(Surv(ttpLmd, e) ~ itCt, data=d1))
(c1 <- coxph(Surv(ttpLmd, e) ~ itCt, data=d1))
```

```
wilcox.test(ttpLmd ~ itCt, data=d1, exact=FALSE)
###
### it + RT + CT
###
(s1 <- survfit(Surv(ttpLmd, e) ~ itRtCt, data=d1))
(c1 <- coxph(Surv(ttpLmd, e) ~ itRtCt, data=d1))
wilcox.test(ttpLmd ~ itRtCt, data=d1, exact=FALSE)
###
### KPS >= 70
###
d1$kGr70 <- ifelse(d1$kpsLmd >= 70, 1, 0)
(s1 <- survfit(Surv(ttpLmd, e) ~ kGr70, data=d1))
(c1 <- coxph(Surv(ttpLmd, e) ~ kGr70, data=d1))
sig(c1)
wilcox.test(ttpLmd ~ kGr70, data=d1, exact=FALSE)
###
###----------------------------------------
### time to death/last observation from LMD
###----------------------------------------
###
### age
###
(s1 <- survfit(Surv(ttlLmd, d) ~ ageLmd, data=d1))
(c1 <- coxph(Surv(ttlLmd, d) ~ ageLmd, data=d1))
###
### gender
###
(s1 <- survfit(Surv(ttlLmd, d) ~ sex, data=d1))
(c1 <- coxph(Surv(ttlLmd, d) ~ sex, data=d1))
###
### path
###
(s1 <- survfit(Surv(ttlLmd, d) ~ pathLmd, data=d1))
(c1 <- coxph(Surv(ttlLmd, d) ~ pathLmd, data=d1))
### GBM vs others
(s1 <- survfit(Surv(ttlLmd, d) ~ lmdGbm, data=d1))
(c1 <- coxph(Surv(ttlLmd, d) ~ lmdGbm, data=d1))
sig(c1)
###
### any Tx effective?
###
(s1 <- survfit(Surv(ttlLmd, d) ~ anyTxLmd, data=d1))
(c1 <- coxph(Surv(ttlLmd, d) ~ anyTxLmd, data=d1))
sig(c1)
###
### i.t. CT
###
(s1 <- survfit(Surv(ttlLmd, d) ~ it, data=d1))
(c1 <- coxph(Surv(ttlLmd, d) ~ it, data=d1))
###
### CT
###
(s1 <- survfit(Surv(ttlLmd, d) ~ ct, data=d1))
(c1 <- coxph(Surv(ttlLmd, d) ~ ct, data=d1))
###
### RT
###
(s1 <- survfit(Surv(ttlLmd, d) ~ rt, data=d1))
(c1 <- coxph(Surv(ttlLmd, d) ~ rt, data=d1))
sig(c1)
autoplot(s1)
```

```
a1 <- autoplot(s1, xlab="Time (months)",
               title="Time to death by use of radiotherapy",
               survSize=1,
               type="CI", alpha=0.8,
               legLabs=c("No", "Yes"),
               legTitle="Radiotherapy",
               legTextSize=15,
               pval=TRUE, pX=0.75, pY=0.75)
autoplot(a1)
jpeg(file="ttdRT")
autoplot(a1)
dev.off()
###
### it + RT
###
(s1 <- survfit(Surv(ttlLmd, d) ~ itRt, data=d1))
(c1 <- coxph(Surv(ttlLmd, d) ~ itRt, data=d1))
###
### RT + CT
###
(s1 <- survfit(Surv(ttlLmd, d) ~ ctRt, data=d1))
(c1 <- coxph(Surv(ttlLmd, d) ~ ctRt, data=d1))
###
### it + CT
###
(s1 <- survfit(Surv(ttlLmd, d) ~ itCt, data=d1))
(c1 <- coxph(Surv(ttlLmd, d) ~ itCt, data=d1))
###
### it + RT + CT
###
(s1 <- survfit(Surv(ttlLmd, d) ~ itRtCt, data=d1))
(c1 <- coxph(Surv(ttlLmd, d) ~ itRtCt, data=d1))
###
### KPS >= 70
###
(s1 <- survfit(Surv(ttlLmd, d) ~ kGr70, data=d1))
(c1 <- coxph(Surv(ttlLmd, d) ~ kGr70, data=d1))
sig(c1)
###
###----------------------------------------
### survival: two-variable models
###----------------------------------------
###
### ttp
###
### ttp: GBM + KPS
xtabs( ~ lmdGbm + kGr70, data=d1)
(s1 <- survfit(Surv(ttpLmd, e) ~ lmdGbm + kGr70, data=d1))
(c1 <- coxph(Surv(ttpLmd, e) ~ lmdGbm + kGr70, data=d1))
sig(c1)
###
### ttp: any Tx
###
xtabs( ~ anyTxLmd + kGr70, data=d1)
(s1 <- survfit(Surv(ttpLmd, e) ~
               anyTxLmd + kGr70, data=d1))
(c1 <- coxph(Surv(ttpLmd, e) ~
             anyTxLmd + kGr70, data=d1))
sig(c1)
autoplot(s1)
###
```

```
### ttp: CT + KPS
###
xtabs( ~ ct + kGr70, data=d1)
(s1 <- survfit(Surv(ttpLmd, e) ~
               ct + kGr70, data=d1))
(c1 <- coxph(Surv(ttpLmd, e) ~ ct + kGr70, data=d1))
sig(c1)
###
### ttd
###
### ttd: GBM + KPS
###
ftable(xtabs( ~ lmdGbm + kGr70 +d, data=d1))
(s1 <- survfit(Surv(ttlLmd, d) ~
               lmdGbm + kGr70, data=d1))
(c1 <- coxph(Surv(ttlLmd, d) ~
             lmdGbm + kGr70, data=d1))
sig(c1)
###
### ttd: any Tx
###
ftable(xtabs( ~ anyTxLmd + kGr70 +d, data=d1))
(s1 <- survfit(Surv(ttlLmd, d) ~
               anyTxLmd + kGr70, data=d1))
(c1 <- coxph(Surv(ttlLmd, d) ~
             anyTxLmd + kGr70, data=d1))
sig(c1)
autoplot(s1)
comp(s1)
a1 <- autoplot(s1, xlab="Time (months)",
               title="Time to death by use of treatment (Tx) and KPS",
               survSize=1,
               type="CI", alpha=0.8,
               legLabs=c("No Tx \nKPS<70", "Some Tx \nKPS<70", "Some Tx \nKPS>=70"),
               legTitle="",
               legTextSize=15, legSize=4,
               pval=TRUE, pX=0.75, pY=0.75)
a1$plot <- a1$plot + annotate("text", x=57, y=0.55,
                              label="Log-rank test \nfor trend p = 1.0")
autoplot(a1, plotHeight=0.7, tabHeight=0.3)
jpeg(file="ttdTxKps.jpg")
autoplot(a1, plotHeight=0.7, tabHeight=0.3)
dev.off()
###
### ttd: RT + KPS
###
ftable(xtabs( ~ rt + kGr70 +d, data=d1))
(s1 <- survfit(Surv(ttlLmd, d) ~ rt + kGr70, data=d1))
(c1 <- coxph(Surv(ttlLmd, d) ~ rt + kGr70, data=d1))
sig(c1)
###
###----------------------------------------
### Tx after initial progression
###----------------------------------------
###
(survfit(Surv(ttlLmd, d) ~ 1, data=d1[d1$anyTxProg==1, ]) )
(t1 <- table(d1$anyTxProg))
s1 <- t1[[1]]
s2 <- sum(f1)
c(s1/s2, binom.confint(s1, s2, conf.level = 0.95, methods="bayes")[-1])
###
```

```
(t1 <- table(d1$noProgLmd))
s1 <- t1[[2]]
s2 <- sum(t1) - t1[[1]]
s2 <- sum(f1)
c(s1/s2, binom.confint(s1, s2, conf.level = 0.95, methods="bayes")[-1])
xtabs( ~ noProgLmd + pathLmd, data=d1)
###
###----------------------------------------
### discussion
###----------------------------------------
###
### overall median ttp and ttd
###
(s1 <- survfit(Surv(ttpLmd, e) ~ 1, data=d1))
(s1 <- survfit(Surv(ttlLmd, d) ~ 1, data=d1))
###
### spinal mets with GBM
###
with(d1, table(lmdGbm, grepl("spine", d1$lmdSpread)))
sum(d1$lmdGbm==1 & grepl("spine", d1$lmdSpread), na.rm=TRUE)
(s1 <- survfit(Surv(ttlLmd, d) ~ 1, data=d1[d1$lmdGbm==1 & grepl("spine", d1$lmdSpread), ]))
###
(s1 <- survfit(Surv(ttpLmd, e) ~ 1, data=d1[d1$lmdGbm==1 & grepl("spine", d1$lmdSpread), ]))
summary(s1)
###
### spinal mets all causes
###
s1 <- survfit(Surv(ttlLmd, d) ~ 1, data=d1[grepl("spine", d1$lmdSpread), ])
###
### use of CT, RT in our series
###
xtabs( ~ grepl("spine", d1$lmdSpread) + ct, data=d1)
xtabs( ~ grepl("spine", d1$lmdSpread) + rt, data=d1)
xtabs( ~ grepl("spine", d1$lmdSpread) + it, data=d1)
###
### Tx with TMZ and RT
###
(s1 <- survfit(Surv(ttpLmd, e) ~ 1,
               data=d1[d1$rt==1 & grepl("TMZ", d1$ctLmd), ]))
(s1 <- survfit(Surv(ttlLmd, d) ~ 1,
               data=d1[d1$rt==1 & grepl("TMZ", d1$ctLmd), ]))
###
### incidence
###
100 * sum(grepl("GBM", d1$pathDx)) / (10 * 60)
###
### spinal cord involvement: ttp & ttd
###
survfit(Surv(ttpLmd, e) ~ 1, data=d1[grepl("spine", d1$lmdSpread), ])
survfit(Surv(ttlLmd, d) ~ 1, data=d1[grepl("spine", d1$lmdSpread), ])
###
### shunt by no. surgeries
###
t1 <- with(d1, table(vps, noSxProg>=1))
fisher.test(t1)
###
### 4th ventricle involved
###
sum(grepl("4V|4th", d1$origLmd)) +
    sum(grepl("4V|4th", d1$lmdSpread))
table(grepl("4V|4th", d1$origLmd) |
```

```
      grepl("4V|4th", d1$lmdSpread),
      d1$naus)
###
### Depocyt
###
sum(grepl("DC", d1$itTx))
survfit(Surv(ttpLmd, e) ~ 1, data=d1[grepl("DC", d1$itTx), ])
table(grepl("DC", d1$itTx),
      d1$vps)
```
